# Gold(III)-Catalyzed Propargylic Substitution Reaction Followed by Cycloisomerization for Synthesis of Poly-Substituted Furans from *N*-Tosylpropargyl Amines with 1,3-Dicarbonyl Compounds

**DOI:** 10.3390/molecules29020378

**Published:** 2024-01-11

**Authors:** Nobuyoshi Morita, Shingo Uchida, Hitomi Chiaki, Naho Ishii, Kentaro Tanikawa, Kosaku Tanaka, Yoshimitsu Hashimoto, Osamu Tamura

**Affiliations:** 1 Department of Pharmacy, Showa Pharmaceutical University, 3-3165 Higashi-Tamagawagakuen, Machida 194-8543, Japan; 2 Research Foundation ITSUU Laboratory, C1232 Kanagawa Science Park R & D Building, 3-2-1 Sakado Takatsu-ku, Kawasaki 213-0012, Japan

**Keywords:** gold catalyst, poly-substituted furans, *N*-tosylpropargyl amines, propargylic substitution, cycloisomerization

## Abstract

The treatment of *N*-tosylpropargyl amines **1** with 1,3-dicarbonyl compounds **2** in the presence of AuBr_3_ (5 mol%) and AgOTf (15 mol%) afforded poly-substituted furans **3** in good-to-high yields via the gold-catalyzed cleavage of the sp^3^ carbon–nitrogen bond.

## 1. Introduction

**Scheme 1:**
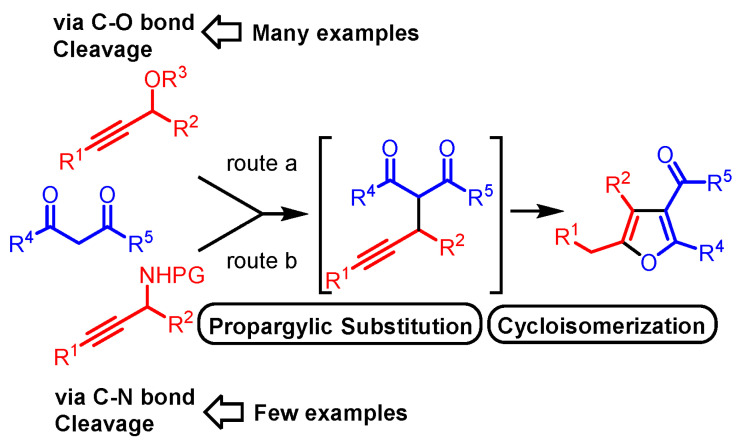
Tandem reaction for synthesis of poly-substituted furans from propargylic alcohols or propargyl amines with 1,3-dicarbonyl compounds.

Poly-substituted furans are valuable structural motifs in both naturally occurring and artificial compounds with a range of biological activities [1,2,3] and are also used as building blocks to synthesize organic compounds [4,5]. Among the numerous procedures reported for the construction of poly-substituted furans [6,7], the tandem reaction of propargyl alcohols, ethers, or acetoxy with active methylene compounds to produce poly-substituted furans via propargylic substitution followed by cycloisomerization has attracted much attention (Scheme 1, route a) [8,9,10]. In the initial propargylic substitution reaction, a hydroxyl, ether, or acetoxy group in the propargylic derivative serves as a leaving group and the reaction proceeds smoothly via the cleavage of the C-O bond to afford propargylic substitution products, which undergo cycloisomerization to yield furans. However, there are few examples of substitution reactions with nucleophiles using nitrogen leaving groups via C-N bond cleavage [11,12,13,14,15,16,17,18,19,20,21], and even fewer examples of propargylic substitution reactions of propargylic amines with active methylene compounds to obtain poly-substituted furans (Scheme 1, route b) [22]. The reason why there are few examples of reactions via C-N bond cleavage is presumably that nitrogen functional groups have inherently low leaving ability, and in addition, the preparation of nitrogen functional groups from other functional groups is cumbersome. However, given the rapid development of organic and biomolecular chemistry in recent years [23], the development of reactions involving C-N bond cleavage is attracting more attention. For example, Tian and co-workers reported the synthesis of poly-substituted furans from *N*-tosylpropargyl amine and active methylene compounds [22]. However, their method requires a high loading of ZnCl_2_ (10 mol%) and TMSCl (50 mol%) and has a narrow scope, with only four examples of the construction of furans reported. Therefore, the development of synthetic methods for the synthesis of poly-substituted furans with cleavage of C-N bonds is a very important topic.

**Scheme 2:**
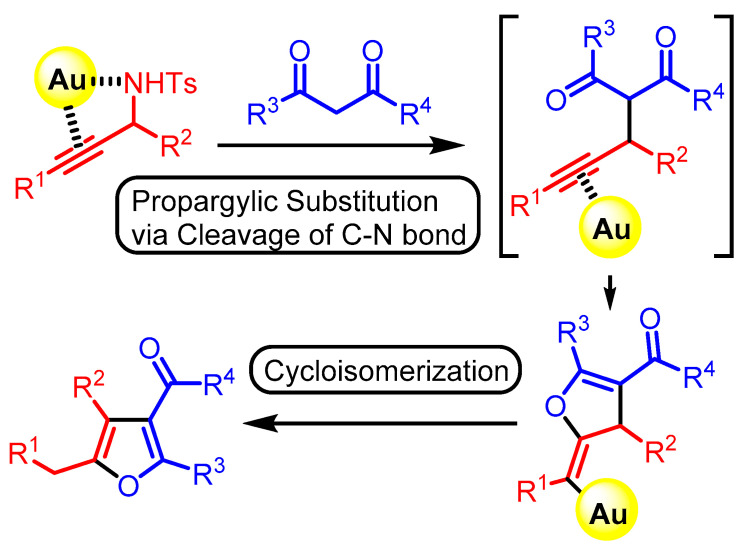
Strategic use of gold catalyst for the synthesis of poly-substituted furans from *N*-tosylpropargyl amines with 1,3-dicarbonyl compounds.

We considered that poly-substituted furans might be synthesized with a propargylic substitution reaction using the NHTs group of *N*-tosylpropargyl amine as a leaving group [11,12,13,14,15,16,17,18,19,20,21,22], followed by cycloisomerization, based on the strategic use of a gold catalyst (Scheme 2). Investigating the reported examples of substitution reactions using nitrogen leaving groups, it was predicted that nitrogen functional groups bearing the tosyl group as a protecting group would have sufficient leaving ability [11,12,13,14,15,16,17,18,19,20,21]. Gold catalysts are expected to promote propargylic substitution reactions by coordinating with the nitrogen atom of the NHTs group and the triple bond in propargyl amine, giving the propargylic substituted product as an intermediate, followed by cycloisomerization mediated by coordination of the gold catalyst with the triple bond to afford poly-substituted furans. The key point is how efficiently the first- and second-step reactions can be carried out with the same gold catalyst. Here, we report a gold-catalyzed propargylic substitution reaction followed by cycloisomerization for the synthesis of poly-substituted furans from *N*-tosylpropargyl amines with active methylene compounds.

## 2. Results

Based on our previous work on the formation of cyclic compounds [24,25,26,27,28,29], we started our study by examining the reaction of *N*-tosylpropargyl amine **1a** as a model substrate with acetylacetone (**2a**) as a nucleophile in the presence of various gold catalysts and silver catalysts in ClCH_2_CH_2_Cl (Table 1).

Treatment of *N*-tosylpropargyl amine **1a** with one equivalent of acetylacetone (**2a**) in the presence of gold(III) catalyst (5 mol% AuBr_3_) at reflux afforded the desired product **3aa** in a 23% yield (entry 1). Increasing the amount of acetylacetone (**2a**) to three equivalents in the reaction slightly improved the yield of the desired product **3aa** (entry 2). The use of 10 equivalents of acetylacetone (**2a**) in the reaction gave a complicated mixture (entry 3). The combined system of a gold(III) catalyst (5 mol% AuBr_3_) and silver catalyst (15 mol% AgOTf) increased the yield of the desired product **3aa** (entry 4). Furthermore, extending the reaction time and increasing the equivalent of acetylacetone (**2a**) improved the yield of the desired product **3aa** (entries 5 and 6). In contrast, the reaction with gold(I) catalyst (5 mol% of AuCl/5 mol% AuCl with 15 mol% AgOTf) afforded the product **3aa** in low yields (entries 7 and 8). The use of a gold(I) catalyst including phosphine ligand Ph_3_PAuCl (5 mol%) in the reaction afforded a trace amount of the desired product **3aa** (entry 9), whereas cationic gold species generated from Ph_3_PAuCl (5 mol%) and AgOTf (5 mol%) gave the desired product **3aa** in a 25% yield (entry 10). The use of a gold(I) catalyst containing carbene ligand, choloro [1,3-bis(2,4,6-trimethylphenyl)imidazol-2-ylidene]gold(I) (5 mol%: NHCAuCl) in the reaction furnished a trace amount of the product **3aa** (entry 11), whereas employing NHCAuCl (5 mol%) and AgOTf (5 mol%) in the reaction afforded the product **3aa** in a low yield (entry 12). These results indicate that 5 mol% AuBr_3_ and 15 mol% AgOTf are a good catalyst system and that the NHTs groups in *N*-tosylpropargyl amine **1a** have sufficient leaving ability in a propargylic substitution reaction.

To examine the effect of the counter anion of the silver co-catalyst, we conducted reactions with various types of silver catalysts, such as AgNTf_2_, AgSbF_6_, AgBF_4_, and AgPF_6_ (Table 2). Treatment of *N*-tosylpropargyl amine **1a** with three equivalents of acetylacetone (**2a**) in the presence of AuBr_3_ (5 mol%) with AgNTf_2_ (15 mol%) or AgSbF_6_ (15 mol%) in ClCH_2_CH_2_Cl at 60 °C for 19 h afforded the desired product **3aa** in moderate yields, 46% or 43%, respectively (entries 2 and 3). The use of AgBF_4_ (15 mol%) in the reaction furnished the desired product **3aa** in a low yield, 24% (entry 4). Employing AgPF_6_ (15 mol%) in the reaction gave no desired product **3aa** (entry 5). These experiments showed that the triflate anion (TfO¯) is effective as a counter anion in the sequential reaction. It has been reported by other groups that the triflate anion (TfO¯) has a good effect as a counter anion in gold-catalyzed reactions [30,31,32]. 

Next, we examined the effect of solvents such as MeNO_2_, toluene, CF_3_CH_2_OH, and CH_3_CN (Table 3). The use of MeNO_2_ in the reaction afforded the desired product **3aa** in a moderate yield, 43% (entry 2). The reaction with toluene or CF_3_CH_2_OH gave only a low yield of the product **3aa**, 33% or 24%, respectively (entries 3 and 4). The use of CH_3_CN in the reaction gave a complex mixture (entry 5). These experiments showed that dichloroethane (ClCH_2_CH_2_Cl) is an effective solvent in the propargylic substitution reaction/cycloisomerization sequences from *N*-tosylpropargyl amine **1a** with acetylacetone (**2a**).

Finally, the optimal reaction conditions for preparation of the desired product **3aa** from *N*-tosylpropargyl amine **1a** with acetylacetone (**2a**) were found to be 5 mol% AuBr_3_ and 15 mol% AgOTf in dichloroethane solvent, stirred at 60 °C for 19 h.

We next investigated the scope and limitations of the gold(III)-catalyzed propargylic substitution reaction followed by cycloisomerization for the synthesis of poly-substituted furans **3** from the *N*-tosylpropargyl amines **1a** and **1b** and various 1,3-dicarbonyl compounds **2a**–**g** (Table 4). The reaction of the *N*-tosylpropargylic amines **1a** and **2b**–**g** gave the corresponding poly-substituted furans **3ab**–**ad** in good-to-high yields, but the corresponding products **3ae**–**3ag** were obtained in moderate yields. Furthermore, the reaction also proceeds with acetylene bearing a phenyl or even a *n*-hexyl group at the terminus, with slightly lower yields in the case of the *n*-hexyl group. It is noteworthy that the reaction of β-keto esters **2c** and **2g** also afforded poly-substituted furans **3** in satisfactory yields. Poly-substituted furans **3aa** (Table 1) and **3ac** (Table 4) were also synthesized by the Tian group [22]. Their reaction conditions were a high loading of ZnCl_2_ (10 mol%) and TMSCl (50 mol%) as reagents in a ClCH_2_CH_2_Cl solvent at 70 °C for a 24 h reaction time. Comparing the yields of the poly-substituted furans **3aa** and **3ac** obtained by our procedure and their procedure, our procedure showed higher yields (**3aa**: 81% vs. 65%/**3ac**: 73% vs. 47%).

**Scheme 3:**
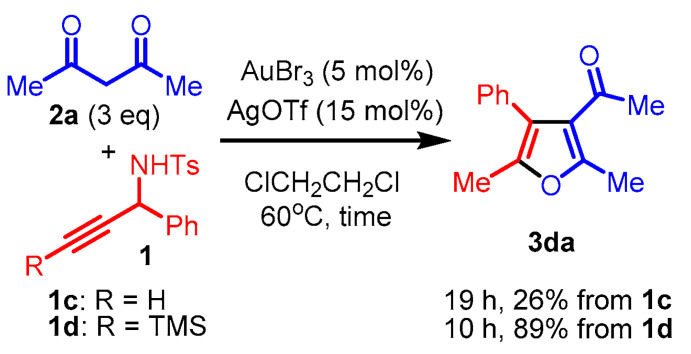
The reaction of *N*-tosylpropargyl amines **1c** (R = H) and **1d** (R = TMS) with acetylacetone (**2a**).

Next, we investigated the reaction of *N*-tosylpropargyl amine **1c** with no substituent at the terminus of acetylene (Scheme 3). The corresponding product **3da** was obtained only in a low yield. On the other hand, when the reaction was carried out with a silyl group-substituted acetylene **1d** instead of no substituted acetylene **1c**, the product **3da** was obtained in a high yield while undergoing desilylation (Scheme 3). Furthermore, it was found that the reaction was faster for substrates with silyl groups than for those with alkyl or aryl groups. This is presumably due to the β-cation-stabilizing effect of the silyl groups [33,34], which would accelerate the cycloisomerization (see Scheme 7).

We next examined the scope and limitations of the reaction with *N*-tosylpropargyl amines **1d** bearing a silyl group at the terminus of acetylene with 1,3-dicarbonyl compounds **2a** and **2d** (Table 5). The reaction proceeded smoothly to afford desilylated products **3da** and **3dd** in 89% and 72% yields, respectively. In addition, the reaction of *N*-tosylpropargyl amine **1e** bearing a 1-naphthyl group at the propargylic position with various 1,3-dicarbonyl compounds **2** was investigated (Table 5). The reaction of *N*-tosylpropargyl amine **1e** with 1,3-dicarbonyl compounds **2a** and **2c** afforded the corresponding furans **3ea** and **3ec** in good-to-high yields (95% and 74%), but the reaction of **1e** with 1,3-dicarbonyl compound **2d** furnished only a low yield of furan **3ea**.

**Scheme 4:**
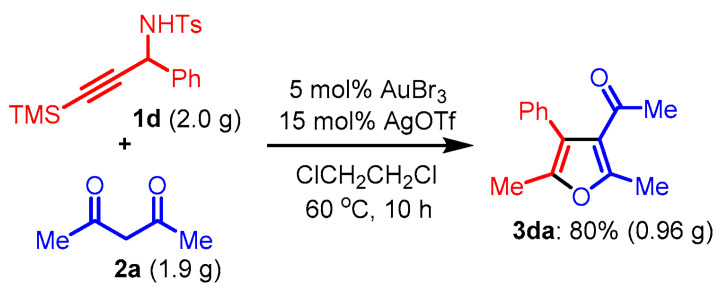
Large-scale preparation of poly-substituted furan **3da** from *N*-tosylpropargyl amine **1d** with acetylacetone (**2a**).

We next conducted a gram-scale preparation of poly-substituted furan **3da** from 2.0 g (5.6 mmol) of *N*-tosylpropargyl amine **1d** and 1.9 g of acetylacetone (**2a**) in the presence of 5 mol% AuBr_3_ and 15 mol% AgOTf in ClCH_2_CH_2_Cl for 10 h at 60 °C, obtaining the product **3da** in an 80% yield (Scheme 4).

**Scheme 5:**
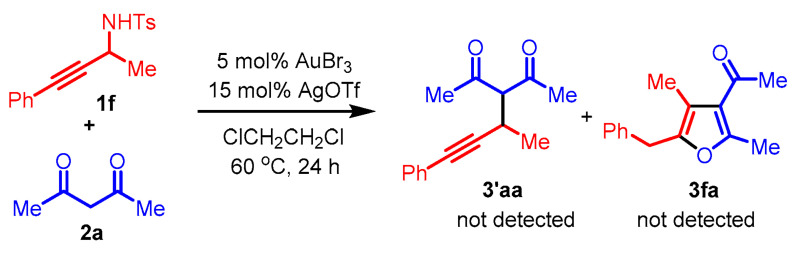
The reaction of *N*-tosylpropargyl amines **1f** bearing a methyl group at the propargylic position with acetylacetone (**2a**).

The reaction of *N*-tosylpropargyl amine **1f** bearing a methyl group at the propargylic position instead of an aryl group afforded neither the propargylic substitution product **3fa** nor the desired product **3aa** (Scheme 5), which shows that stabilization of the cation at the propargylic position by the aryl group (phenyl or 1-naphthyl) in *N*-tosylpropargyl amine **1** is very important for the step of propargylic substitution reaction (see Scheme 7).

**Scheme 6:**
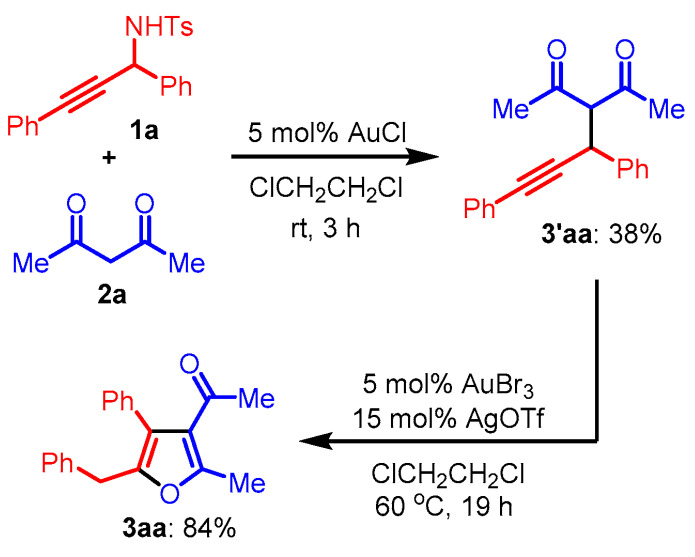
Generation of intermediate **3′aa** leading to poly-substituted furan **3aa**.

To confirm the reaction pathway, the reaction of *N*-tosylpropargyl amine **1a** and acetylacetone (**2a**) was conducted in the presence of 5 mol% of AuCl in ClCH_2_CH_2_Cl at room temperature for 3 h, affording the propargylic substitution product **3′aa** in a 38% yield (Scheme 6). Moreover, **3′aa** was smoothly converted into the corresponding poly-substituted furan **3aa** in an excellent yield in the presence of 5 mol% of AuBr_3_ and 15 mol% AgOTf at 60 °C in ClCH_2_CH_2_Cl. These experiments clearly indicated that the reaction proceeds via a propargylic substitution reaction [34].

**Scheme 7:**
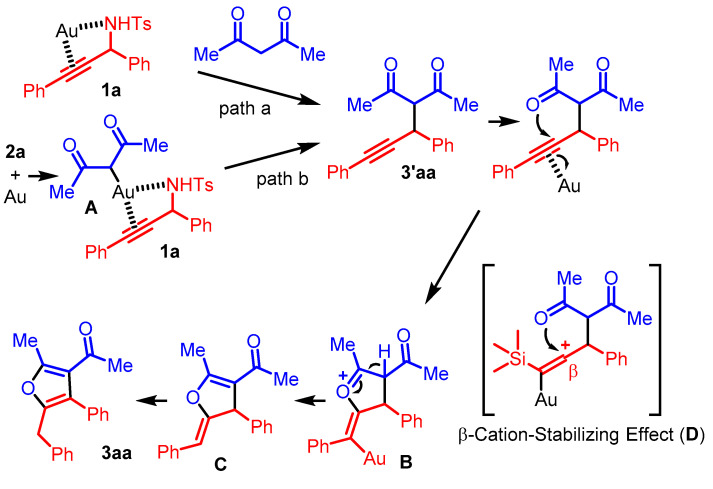
Plausible mechanism of gold(III)-catalyzed propargylic substitution reaction followed by cycloisomerization leading to poly-substituted furan **3aa**.

A plausible mechanism of the gold(III)-catalyzed propargylic substitution reaction followed by cycloisomerization leading to poly-substituted furan **3aa** is shown in Scheme 7. We considered two possible pathways for the propargylic substitution reaction of *N*-tosylpropargyl amine **1a** with acetylacetone (**2a**). First, the gold catalyst would coordinate with the triple bond and the nitrogen atom in **1a**, to promote the propargylic substitution reaction of acetylacetone (**2a**) (Scheme 7, path a). Second, the propargylic substitution reaction of the gold enolate species **A** [35,36,37,38,39,40,41,42,43] generated by the reaction of the gold(III) catalyst and acetylacetone (**2a**) would occur via coordination of the triple bond and the nitrogen atom of **1a** (Scheme 7, path b), although it still remains unclear whether the gold(III)-enolate species **A** is generated in this gold-catalyzed reaction. After completion of the propargylic substitution reaction, the gold catalyst would coordinate to the triple bond of **3′aa** to enable smooth cyclization, affording the vinyl gold complex **B**. Deauration and isomerization would afford the furan **3aa** (**B**→**C**→**3aa**). When the substituent on the triple bond of *N*-tosylpropargyl amines **1** is a silyl group, the reaction would be accelerated by the β-cation-stabilizing effect **D** of the silyl group [33,34]. This may explain why the reaction of *N*-tosylpropargyl amines **1d** and **1e** bearing a silyl group proceeds faster than that of *N*-tosylpropargyl amines **1** with alkyl or aryl substituents on the triple bond.

## 3. Materials and Methods

### 3.1. General Information

^1^H and ^13^C NMR spectra were recorded with a JEOL JNM-AL300 (Japan Electron Optics Laboratory Co., Ltd., Tokyo, Japan) or BRUKER AV-300 spectrometer (Bruker, Billerica, MA, USA) at room temperature, with tetramethylsilane as an internal standard (CDCl_3_ solution). Chemical shifts were recorded in ppm and coupling constants (*J*) in Hz. Infrared (IR) spectra were recorded with a Shimadzu FTIR-8200A spectrometer (Shimadzu Corporation, Kyoto, Japan). Mass spectra were recorded on JEOL JMS-700 spectrometers (Japan Electron Optics Laboratory Co., Ltd., Tokyo, Japan). Merck silica gel 60 (1.09385) (Merck, Darmstadt, Germany) and Merck silica gel 60 F254 (Merck, Darmstadt, Germany) were used for column chromatography and thin-layer chromatography (TLC), respectively.

### 3.2. General Procedure for Gold(III)-Catalyzed Propargylic Substitution Reaction Followed by Cycloisomerization for Synthesis of Poly-Substituted Furans 3 from N-Tosylpropargyl Amines 1 with 1,3-Dicarbonyl Compounds **2**

Five mol% AuBr_3_ and fifteen mol% AgOTf were added at room temperature to a solution of *N*-tosylpropargyl amine **1** and 1,3-dicarbonyl compound **2** in ClCH_2_CH_2_Cl, and the mixture was stirred at 60 °C. After the complete consumption of *N*-tosylpropargyl amine **1** (the reaction was monitored by thin-layer chromatography; usually within 19 h for *N*-tosylpropargyl amines **1a** and **1b**, and within 10 h for *N*-tosylpropargyl amines **1d** and **1e**), the solvent was removed in vacuo and the crude product was subjected to SiO_2_ column chromatography (eluents = hexane:AcOEt or diethyl ether) to give poly-substituted furan **3**.

*1-(5-Benzyl-2-methyl-4-phenylfuran-3-yl)ethan-1-one* (**3aa**); colorless oil, yield = 81%, 33 mg (hexane:AcOEt = 10:1), IR (KBr) 3030, 1672, 1558 cm^−1^; ^1^H-NMR (300 MHz, CDCl_3_) δ 7.41–7.36 (4H, m), 7.29–7.23 (4H, m), 7.14–7.12 (2H, m), 3.81 (2H, s), 2.53 (3H, s), 1.92 (3H, s); ^13^C-NMR (75 MHz, CDCl_3_) δ 196.0, 157.2, 148.8, 138.1, 133.4, 130.0, 128.5, 128.3, 127.6, 126.4, 122.9, 121.9, 32.0, 30.7, 14.4 (overlapped); HRMS (EI) *m*/*z* calcd for C_20_H_18_O_2_ 290.1307, found 290.1311.

*1-(5-Benzyl-2-ethyl-4-phenylfuran-3-yl)propan-1-one* (**3ab**); colorless oil, yield = 70%, 31 mg (hexane:diethyl ether = 10:1), IR (KBr) 3030, 1672, 1558 cm^−1^; ^1^H-NMR (300 MHz, CDCl_3_) δ 7.43–7.35 (4H, m), 7.33–7.24 (4H, m), 7.14–7.11 (2H, m), 3.82 (2H, s), 2.91 (2H, q, *J* = 7.2 Hz), 2.16 (2H, q, *J* = 7.5 Hz), 1.25 (3H, t, *J* = 7.5 Hz), 0.90 (3H, t, *J* = 7.2 Hz); ^13^C-NMR (75 MHz, CDCl_3_) δ 199.4, 161.4, 148.6, 138.2, 133.6, 129.9, 128.5, 128.3, 127.5, 127.2, 126.4, 121.9, 121.5, 35.8, 32.0, 21.5, 12.4, 8.0; HRMS (EI) *m*/*z* calcd for C_22_H_22_O_2_ 318.1620, found 318.1620.

*Ethyl 5-benzyl-2-methyl-4-phenylfuran-3-carboxylate* (**3ac**); colorless oil, yield = 73%, 31 mg (hexane:diethyl ether = 10:1), IR (KBr) 2990, 1708, 1602 cm^−1^; ^1^H-NMR (300 MHz, CDCl_3_) δ 7.38–7.32 (4H, m), 7.30–7.22 (4H, m), 7.15–7.12 (2H, m), 4.10 (2H, q, *J* = 6.9 Hz), 3.85 (2H, s), 2.56 (3H, s), 1.08 (3H, t, *J* = 6.9 Hz); ^13^C-NMR (75 MHz, CDCl_3_) δ 164.2, 158.3, 148.8, 138.3, 133.0, 130.0, 128.5, 128.4, 127.7, 127.0, 126.4, 122.7, 113.6, 59.8, 32.0, 14.2, 13.9; HRMS (EI) *m*/*z* calcd for C_21_H_20_O_3_ 320.1412, found 320.1417.

The ^1^H-NMR and ^13^C-NMR data are identical to the reported values [22].

*Ethyl 5-benzyl-2,4-diphenylfuran-3-carboxylate* (**3ad**); colorless oil, yield = 81%, 57 mg (hexane:diethyl AcOEt = 30:1), IR (KBr) 1718 cm^−1^; ^1^H-NMR (300 MHz, CDCl_3_) δ 7.81 (2H, dd, *J* = 6.6, 1.5 Hz), 7.59–7.33 (5H, m), 7.28–7.26 (4H, m), 7.25–7.19 (4H, m), 4.08 (2H, q, *J* = 6.9 Hz), 3.98 (2H, s), 0.97 (3H, t, *J* = 6.9 Hz); ^13^C-NMR (75 MHz, CDCl_3_) δ 164.5, 156.5, 154.7, 150.0, 138.0, 132.7, 130.0, 129.7, 129.0, 128.9, 128.6, 128.4, 128.1, 128.0, 127.7, 127.3, 126.5, 60.5, 32.3, 13.6; HRMS (EI) *m*/*z* calcd for C_26_H_22_O_3_ 382.1569, found 382.1570.

*Methyl 5-benzyl-4-phenyl-2-propylfuran-3-carboxylate* (**3ae**); colorless oil, yield = 53%, 25 mg (hexane:AcOEt = 20:1), IR (KBr) 3030, 1724 cm^−1^; ^1^H-NMR (300 MHz, CDCl_3_) δ 7.37–7.33 (4H, m), 7.30–7.20 (4H, m), 7.14–7.10 (2H, m), 3.86 (2H, s), 3.64 (3H, s), 2.94 (2H, t, *J* = 7.5 Hz), 1.71 (2H, m), 0.96 (3H, t, *J* = 7.2 Hz); ^13^C-NMR (75 MHz, CDCl_3_) δ 164.7, 162.2, 148.9, 138.3, 133.0, 129.9, 128.5, 128.3, 127.8, 127.0, 126.4, 122.5, 113.0, 50.9, 32.0, 29.9, 21.6, 13.8; HRMS (EI) *m*/*z* calcd for C_22_H_22_O_3_ 334.1569, found 334.1566.

*Methyl 5-benzyl-2-butyl-4-phenylfuran-3-carboxylate* (**3af**); colorless oil, yield = 55%, 27 mg (hexane:AcOEt = 20:1), IR (KBr) 3030, 1714 cm^−1^; ^1^H-NMR (300 MHz, CDCl_3_) δ 7.39–7.32 (4H, m), 7.30–7.21 (4H, m), 7.18–7.11 (2H, m), 3.86 (2H, s), 3.63 (3H, s), 2.96 (2H, t, *J* = 7.2 Hz), 1.71–1.59 (2H, m), 1.43–1.30 (2H, m), 0.92 (3H, t, *J* = 7.5 Hz); ^13^C-NMR (75 MHz, CDCl_3_) δ 164.7, 162.4, 148.9, 138.3, 132.9, 129.9, 128.5, 128.3, 127.7, 127.0, 126.3, 122.5, 112.9, 50.9, 32.0, 30.2, 28.0, 22.3, 13.8; HRMS (EI) *m*/*z* calcd for C_23_H_24_O_3_ 348.1725, found 348.1723.

*Methyl 2,5-dibenzyl-4-phenylfuran-3-carboxylate* (**3ag**); colorless oil, yield = 42%, 23 mg (hexane:AcOEt = 20:1), IR (KBr) 2923, 1710 cm^−1^; ^1^H-NMR (300 MHz, CDCl_3_) δ 7.38–7.09 (13H, m), 7.16–7.10 (2H, m), 4.32 (2H, s), 3.85 (2H, s), 3.65, (3H, s); ^13^C-NMR (75 MHz, CDCl_3_) δ 164.4, 159.5, 149.8, 137.6, 132.7, 130.0, 128.8, 128.52, 128.47, 128.40, 128.3, 127.8, 127.1, 126.5, 126.4, 126.3, 122.7, 51.0, 33.9, 32.0; HRMS (EI) *m*/*z* calcd for C_26_H_22_O_3_ 382.1569, found 382.1571.

*1-(5-Heptyl-2-methyl-4-phenylfuran-3-yl)ethan-1-one* (**3ba**); colorless oil, yield = 66%, 19 mg (hexane:AcOEt = 20:1), IR (KBr) 2956, 1676 cm^−1^; ^1^H-NMR (300 MHz, CDCl_3_) δ 7.43–7.33 (3H, m), 7.25–7.22 (2H, m), 2.54 (3H, s), 2.45 (2H, t, *J* = 7.2 Hz), 1.90 (3H, s), 1.56 (2H, br t, *J* = 7.2 Hz), 1.25–1.21 (8H, m), 0.86 (3H, t, *J* = 6.9 Hz); ^13^C-NMR (75 MHz, CDCl_3_) δ 196.2, 156.3, 151.1, 133.8, 130.0, 128.4, 127.3, 122.9, 120.6, 31.7, 30.7, 29.0, 28.9, 28.5, 25.7, 22.6, 14.3, 14.0; HRMS (EI) *m*/*z* calcd for C_20_H_26_O_2_ 298.1933, found 298.1933.

*1-(2-Ethyl-5-heptyl-4-phenylfuran-3-yl)propan-1-one* (**3bb**); colorless oil, yield = 61%, 22 mg (hexane:AcOEt = 20:1), IR (KBr) 2956, 1697 cm^−1^; ^1^H-NMR (300 MHz, CDCl_3_) δ 7.42–7.33 (3H, m), 7.26–7.21 (2H, m), 2.92 (2H, q, *J* = 7.5 Hz), 2.46 (2H, t, *J* = 7.5 Hz), 2.14 (2H, t, *J* = 7.2 Hz), 1.60–1.55 (2H, m), 1.27 (3H, t, *J* = 7.2 Hz), 1.29–1.20 (8H, m), 0.89 (3H, t, *J* = 7.2 Hz), 0.86 (3H, t, *J* = 6.9 Hz); ^13^C-NMR (75 MHz, CDCl_3_) δ 199.7, 160.6, 151.1, 134.1, 129.9, 128.4, 127.3, 121.8, 120.2, 35.8, 31.7, 29.0, 28.9, 28.5, 25.8, 22.6, 21.5, 14.1, 12.4, 8.1; HRMS (EI) *m*/*z* calcd for C_22_H_30_O_2_ 326.2246, found 326.2236.

*Ethyl 5-heptyl-2-methyl-4-phenylfuran-3-carboxylate* (**3bc**); colorless oil, yield = 53%, 19 mg (hexane:AcOEt = 20:1), IR (KBr) 2956, 1708 cm^−1^; ^1^H-NMR (300 MHz, CDCl_3_) δ 7.37–7.30 (3H, m), 7.24–7.21 (2H, m), 4.09 (2H, q, *J* = 6.9 Hz), 2.58 (3H, s), 2.59 (2H, t, *J* = 7.5 Hz), 1.57–1.53 (2H, m), 1.26–1.20 (8H, m), 1.07 (3H, t, *J* = 7.2 Hz), 0.86 (3H, t, *J* = 6.9 Hz); ^13^C-NMR (75 MHz, CDCl_3_) δ 164.4, 157.4, 151.3, 133.4, 130.0, 127.5, 126.7, 121.2, 113.5, 59.7, 31.7, 29.0, 28.9, 28.5, 25.8, 22.6, 14.1, 14.0, 13.9; HRMS (EI) *m*/*z* calcd for C_21_H_28_O_3_ 328.2038, found 328.2030.

*Ethyl 5-heptyl-2,4-diphenylfuran-3-carboxylate* (**3bd**); colorless oil, yield = 75%, 36 mg (hexane:AcOEt = 20:1), IR (KBr) 2955, 1718 cm^−1^; ^1^H-NMR (300 MHz, CDCl_3_) δ 7.86–7.83 (2H, m), 7.45–7.27 (8H, m), 4.09 (2H, q, *J* = 6.9 Hz), 2.62 (2H, t, *J* = 7.5 Hz), 1.68–1.62 (2H, m), 1.26–1.20 (8H, m), 0.97 (3H, t, *J* = 6.9 Hz), 0.86 (3H, t, *J* = 6.9 Hz); ^13^C-NMR (75 MHz, CDCl_3_) δ 164.7, 153.9, 152.5, 133.1, 130.2, 129.7, 128.7, 128.1, 127.9, 127.6, 127.0, 122.8, 114.7, 60.4, 31.7, 29.1, 28.9, 28.4, 26.0, 22.6, 14.1, 13.6; HRMS (EI) *m*/*z* calcd for C_26_H_30_O_3_ 390.2195, found 390.2189.

*Methyl 5-heptyl-4-phenyl-2-propylfuran-3-carboxylate* (**3be**); colorless oil, yield = 33%, 15 mg (hexane:AcOEt = 20:1), IR (KBr) 2957, 1732 cm^−1^; ^1^H-NMR (300 MHz, CDCl_3_) δ 7.38–7.26 (3H, m), 7.25–7.21 (2H, m), 3.62 (3H, s), 2.95 (2H, t, *J* = 7.2 Hz), 2.50 (2H, t, *J* = 7.2 Hz), 1.77–1.70 (2H, m), 1.58–1.54 (2H, m), 1.25–1.19 (8H, m), 0.99 (3H, t, *J* = 7.2 Hz), 0.86 (3H, t, *J* = 6.9 Hz); ^13^C-NMR (75 MHz, CDCl_3_) δ 164.9, 161.4, 151.4, 133.4, 129.9, 127.6, 126.7, 121.0, 112.8, 50.8, 31.7, 29.9, 29.0, 28.9, 28.4, 25.8, 22.6, 21.6, 14.1, 13.8; HRMS (EI) *m*/*z* calcd for C_22_H_30_O_3_ 342.2195, found 342.2195.

*1-(2,5-Dimethyl-4-phenylfuran-3-yl)ethan-1-one* (**3da**); colorless oil, yield = 89%, 27 mg (hexane:AcOEt = 10:1), ^1^H-NMR (300 MHz, CDCl_3_) δ 7.40–7.34 (3H, m), 7.26–7.23 (2H, m), 2.53 (3H, s), 2.16 (3H, s), 1.93 (3H, s); ^13^C-NMR (75 MHz, CDCl_3_) δ 196.2, 156.2, 146.9, 133.7, 129.9, 128.4, 127.3, 123.0, 120.8, 30.7, 14.2, 11.6.

The ^1^H-NMR and ^13^C-NMR data are identical to the reported values [44].

*Ethyl 2,5-dimethyl-4-phenylfuran-3-carboxylate* (**3dd**); colorless oil, yield = 72%, 25 mg (hexane:AcOEt = 10:1), ^1^H-NMR (300 MHz, CDCl_3_) δ 7.38–7.23 (4H, m), 4.12 (2H, q, *J* = 7.2 Hz), 2.57 (3H, s), 2.20 (3H, s), 1.09 (3H, t, *J* = 7.2 Hz); ^13^C-NMR (75 MHz, CDCl_3_) δ 164.3, 157.4, 147.1, 133.3, 130.0, 127.6, 126.7, 121.3, 113.5, 59.7, 14.1, 13.9, 11.7.

The ^1^H-NMR and ^13^C-NMR data are identical to the reported values [9].

*1-[2,5-Dimethyl-4-(naphthalen-1-yl)furan-3-yl]ethan-1-one* (**3ea**); colorless oil, yield = 95%, 31 mg (hexane:AcOEt = 15:1), IR (KBr) 1671, 1560, 1507, 1308 cm^−1^; ^1^H-NMR (300 MHz, CDCl_3_) δ 7.92–7.87 (2H, m), 7.69 (1H, dd, *J* = 8.1, 0.9 Hz), 7.55–7.41 (3H, m), 7.38 (1H, dd, *J* = 7.2, 1.5 Hz), 2.64 (3H, s), 2.06 (3H, s), 1.58 (3H, s); ^13^C-NMR (75 MHz, CDCl_3_) δ 195.9, 157.1. 147.8, 133.7, 133.0, 131.4, 128.4, 128.38, 128.27, 128.22, 126.6, 126.1, 125.6, 125.5, 118.4, 29.9, 14.6, 11.7; HRMS (EI) *m*/*z* calcd for C_18_H_16_O_2_ 264.1150, found 264.1153.

*Ethyl 2,5-dimethyl-4-(naphthalen-1-yl)furan-3-carboxylate* (**3ec**); colorless oil, yield = 74%, 27 mg (hexane:AcOEt = 20:1), IR (KBr) 1707, 1596, 1310 1201, 1085 cm^−1^; ^1^H-NMR (300 MHz, CDCl_3_) δ 7.87–7.81 (2H, m), 7.67–7.64 (1H, m), 7.50–7.35 (3H, m), 7.30 (1H, dd, *J* = 7.2, 1.5 Hz), 3.76 (2H, q, *J* = 7.2 Hz), 2.66 (3H, s), 2.11 (3H, s), 0.50 (3H, t, *J* = 7.2 Hz); ^13^C-NMR (75 MHz, CDCl_3_) δ 164.2, 157.7, 147.8, 133.4, 133.2, 131.6, 128.0, 127.6, 126.0, 125.6, 125.5, 125.1, 119.1, 114.9, 59.4, 14.0, 13.1, 11.7 (overlapped); HRMS (EI) *m*/*z* calcd for C_19_H_18_O_3_ 294.1256, found 294.1255.

*Ethyl 5-methyl-4-(naphthalen-1-yl)-2-phenylfuran-3-carboxylate* (**3ed**); colorless oil, yield = 38%, 33 mg (hexane:AcOEt = 20:1), IR (KBr) 1712, 1492, 1374, 1312, 1207, 1112, 1097 cm^−1^; ^1^H-NMR (300 MHz, CDCl_3_) δ 7.99–7.95 (2H, m), 7.90–7.84 (2H, m), 7.77–7.74 (1H, m), 7.53–7.67 (7H, m), 3.74 (2H, q, *J* = 7.2 Hz), 2.23 (3H, s), 0.41 (3H, t, *J* = 7.2 Hz); ^13^C-NMR (75 MHz, CDCl_3_) δ 164.2, 155.0, 149.2, 133.5, 133.1, 131.2, 130.1, 128.9, 128.2, 127.9, 127.7, 127.6, 125.9, 125.8, 125.6, 125.2, 121.2, 115.8, 60.0, 12.9, 12.0 (overlapped); HRMS (EI) *m*/*z* calcd for C_24_H_20_O_3_ 356.1412, found 356.1411.

*3-(1,3-Diphenylprop-2-yn-1-yl)pentane-2,4-dione* (**3′aa**); To a solution of *N*-tosylpropargyl amine **1a** (50 mg, 0.14 mmol) and acetylacetone (**2a**) (42 mg, 0.42 mmol) in ClCH_2_CH_2_Cl (2 mL), AuCl (1.6 mg, 0.0071 mmol, 5 mol%) was added at room temperature. The reaction mixture was stirred at 60 °C for 3 h (the reaction was monitored by thin-layer chromatography). After the complete consumption of *N*-tosylpropargyl amine **1a**, the solvent was removed in vacuo and the crude product was subjected to SiO_2_ column chromatography (hexane:diethyl ether = 10:1) to give the propargylic substitution product **3′aa** (15 mg, 38%) as a colorless oil. ^1^H-NMR (300 MHz, CDCl_3_) δ 7.50–7.26 (10H, m), 4.67 (1H, d, *J* = 11.1 Hz), 4.21 (1H, d, *J* = 11.1 Hz), 2.39 (3H, s), 1.93 (3H, s); ^13^C-NMR (75 MHz, CDCl_3_) δ 201.65, 201.60, 138.2, 131.6, 128.9, 128.34, 128.25, 128.1, 127.8, 122.7, 88.0, 84.9, 75.7, 38.1, 31.1, 28.7.

The ^1^H-NMR and ^13^C-NMR data are identical to the reported values [45].

## 4. Conclusions

In conclusion, we present a gold(III)-catalyzed propargylic substitution reaction followed by cycloisomerization for the synthesis of poly-substituted furans **3** from *N*-tosylpropargyl amines **1** with 1,3-dicarbonyl compounds **2**. The reaction proceeds via the propargylic substitution product intermediate **3′aa**. We are currently applying this method to the synthesis of biologically active cyclic ether derivatives. Experimental and theoretical investigations on the reaction mechanism are also in progress.

## Figures and Tables

**Table 1:**
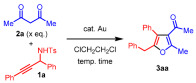
Optimization of the reaction conditions for gold(III)-catalyzed propargylic substitution reaction followed by cycloisomerization.

Entry	Cat. Au (mol%)	x Eq.	Temp.	Time	3aa Yield
1	AuBr_3_ (5)	1	reflux	4 h	23%
2	AuBr_3_ (5)	3	reflux	7 h	33%
3	AuBr_3_ (5)	10	reflux	7 h	complex mixture
4	AuBr_3_ (5)/AgOTf (15)	1	60 °C	5 h	63%
5	AuBr_3_ (5)/AgOTf (15)	3	60 °C	10 h	71%
6	AuBr_3_ (5)/AgOTf (15)	3	60 °C	19 h	81%
7	AuCl (5)	3	60 °C	19 h	7%
8	AuCl (5)/AgOTf (15)	3	60 °C	19 h	26%
9	Ph_3_PAuCl (5)	3	60 °C	19 h	trace
10	Ph_3_PAuCl (5)/AgOTf (5)	3	60 °C	19 h	25%
11	NHCAuCl (5)	3	60 °C	19 h	trace
12	NHCAuCl (5)/AgOTf (5)	3	60 °C	19 h	22%

**Table 2:**
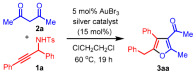
Effect of the counter anion of silver catalyst in gold(III)-catalyzed propargylic substitution reaction followed by cycloisomerization.

Entry	Silver Catalyst	Yield
1	AgOTf	81%
2	AgNTf_2_	46%
3	AgSbF_6_	43%
4	AgBF_4_	24%
5	AgPF_6_	no reaction

**Table 3:**
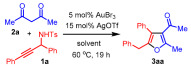
Solvent effect in gold(III)-catalyzed propargylic substitution reaction followed by cycloisomerization.

Entry	Solvent	Yield
1	ClCH_2_CH_2_Cl	81%
2	MeNO_2_	43%
3	Toluene	33%
4	CF_3_CH_2_OH	24%
5	CH_3_CN	complex mixture

**Table 4:**
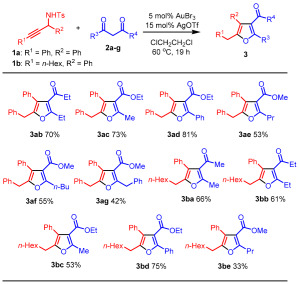
Scope and limitations of the gold(III)-catalyzed propargylic substitution reaction followed by cycloisomerization of N-tosylpropargyl amines **1a** and **1b** with 1,3-dicarbonyl compounds **2a**–**g**.

**Table 5:**
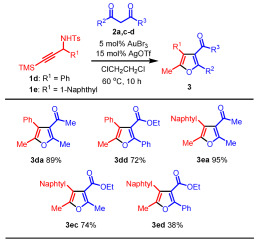
Scope and limitations of the gold(III)-catalyzed propargylic substitution reaction followed by cycloisomerization of N-tosylpropargyl amines **1d** and **1e** with 1,3-dicarbonyl compounds **2**.

## Data Availability

Data are contained within the article and Supplementary Materials.

## Supplementary Materials

The following supporting information can be downloaded at: https://www.mdpi.com/article/10.3390/molecules29020378/s1, ^1^H, ^13^C-NMR spectrum.
