# Supplementary material for: Gold(III)-Catalyzed Propargylic Substitution Reaction Followed by Cycloisomerization for Synthesis of Poly-Substituted Furans from N-Tosylpropargyl Amines with 1,3-Dicarbonyl Compounds
Source: Molecules. 2024 Jan 11;29(2):378. doi: 10.3390/molecules29020378 (PMC10819120; doi:10.3390/molecules29020378)

20161011_NO72

¥¥192.168.226.211¥anal¥5年生¥内田¥20161011_NO721NON_E1.als

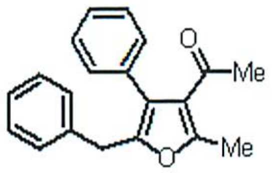

3aa

DFILE	20161011_NO721NON_E1.als
COMNT	20161011_NO72
DATIM	Tue Oct 11 14:17:44 2016
OBNUC	1H
EXMOD	NON
OBFRQ	300.40 MHz
OBSET	130.00 KHz
OBFIN	1150.00 Hz
POINT	16384
FREQU	6006.01 Hz
SCANS	8
ACQTM	2.7279 sec
PD	4.2720 sec
PW1	6.00 usec
IRNUC	1H
CTEMP	22.1 c
SLVNT	CDCL3
EXREF	0.00 ppm
BF	0.12 Hz
RGAIN	22

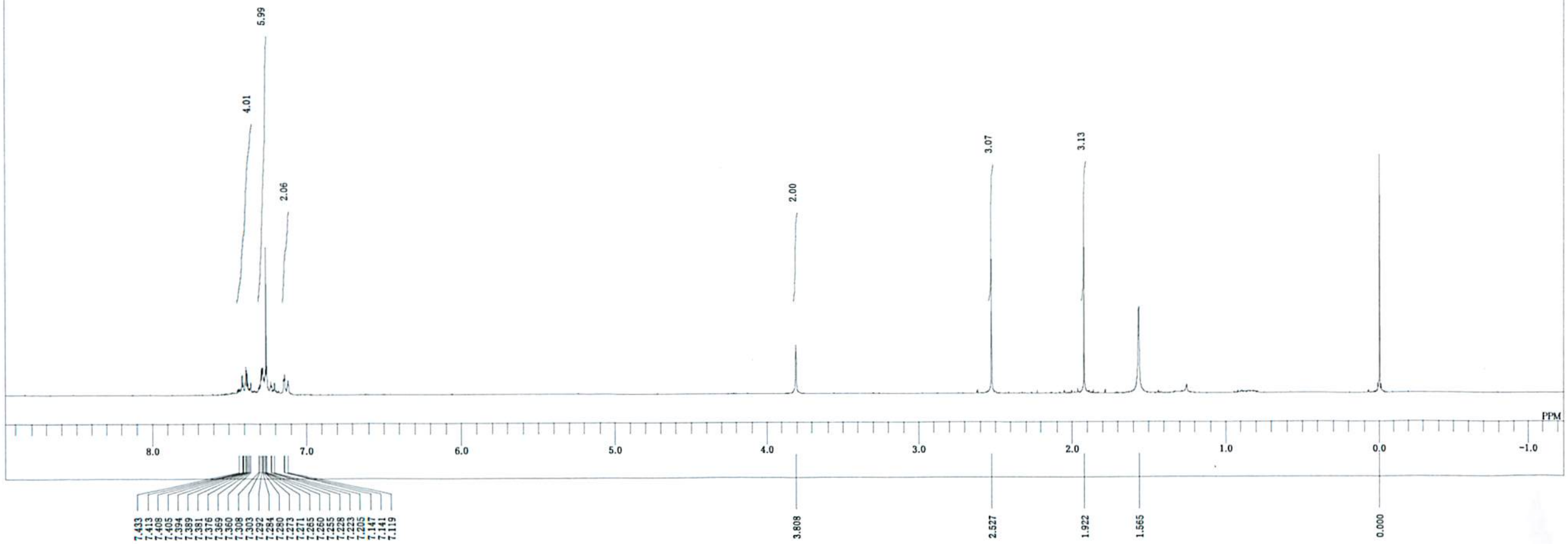

20161012_NO72

¥¥192.168.226.21¥Yenal¥5年生¥内田¥20161012_NO721BCM_E5.als

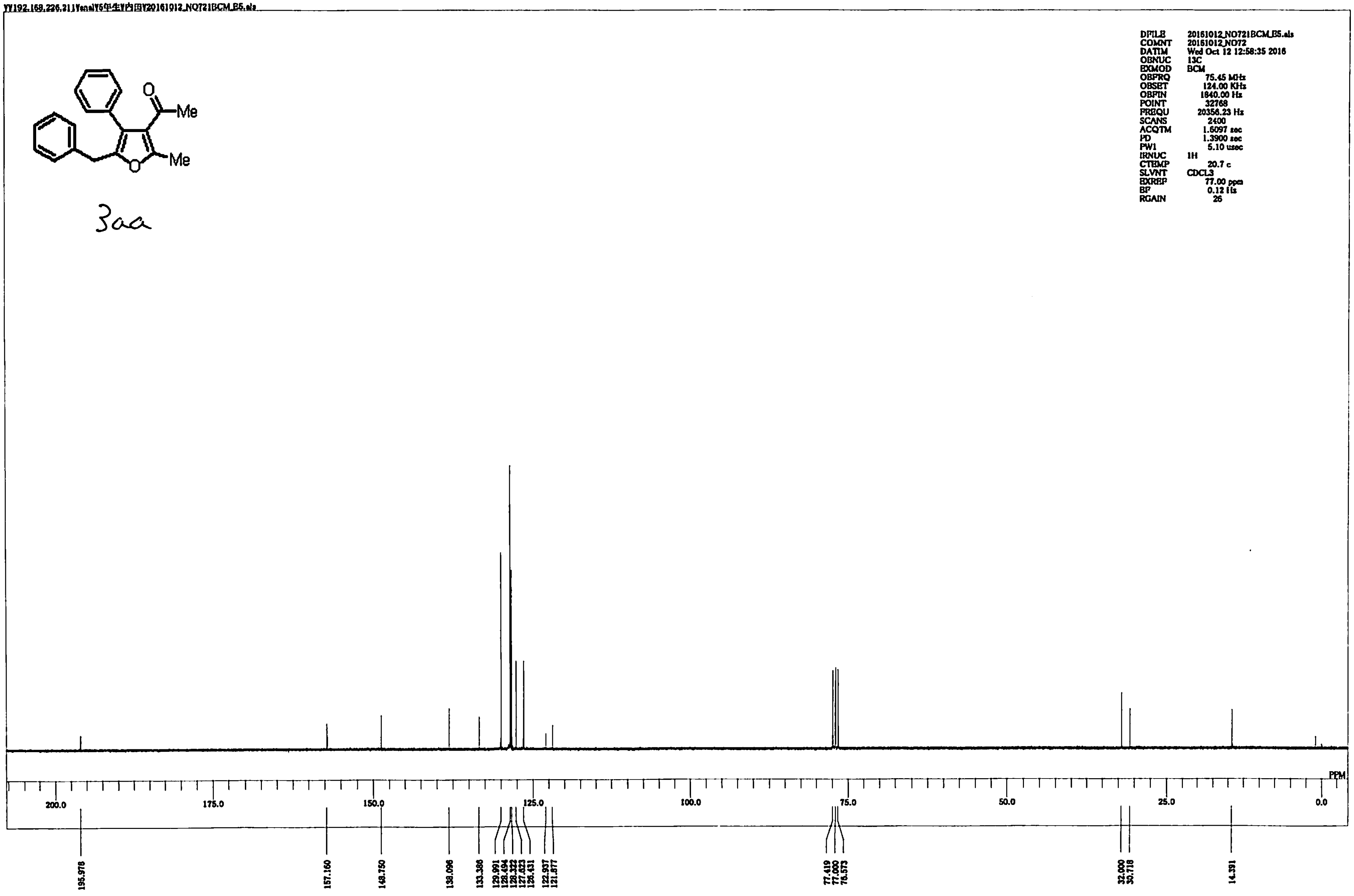

20161124_NO76

\\192.168.226.211\Yana\[illegible]\20161124_NO761NON_E30.als

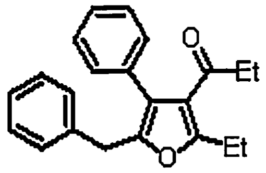

3ab

DFILE	20161124_NO761NON_E30.als
COMNT	20161124_NO76
DATIM	Thu Nov 24 16:10:23 2016
OBNUC	1H
EXMOD	NON
OBFRQ	300.40 MHz
OBSET	130.00 KHz
OBFIN	1150.00 Hz
POINT	16384
FREQU	6006.01 Hz
SCANS	8
ACQTM	2.7279 sec
PD	4.2720 sec
PW1	6.00 usec
IRNUC	1H
CTEMP	21.5 c
SLVNT	CDCL3
EXREF	0.00 ppm
BF	0.12 Hz
RGAIN	20

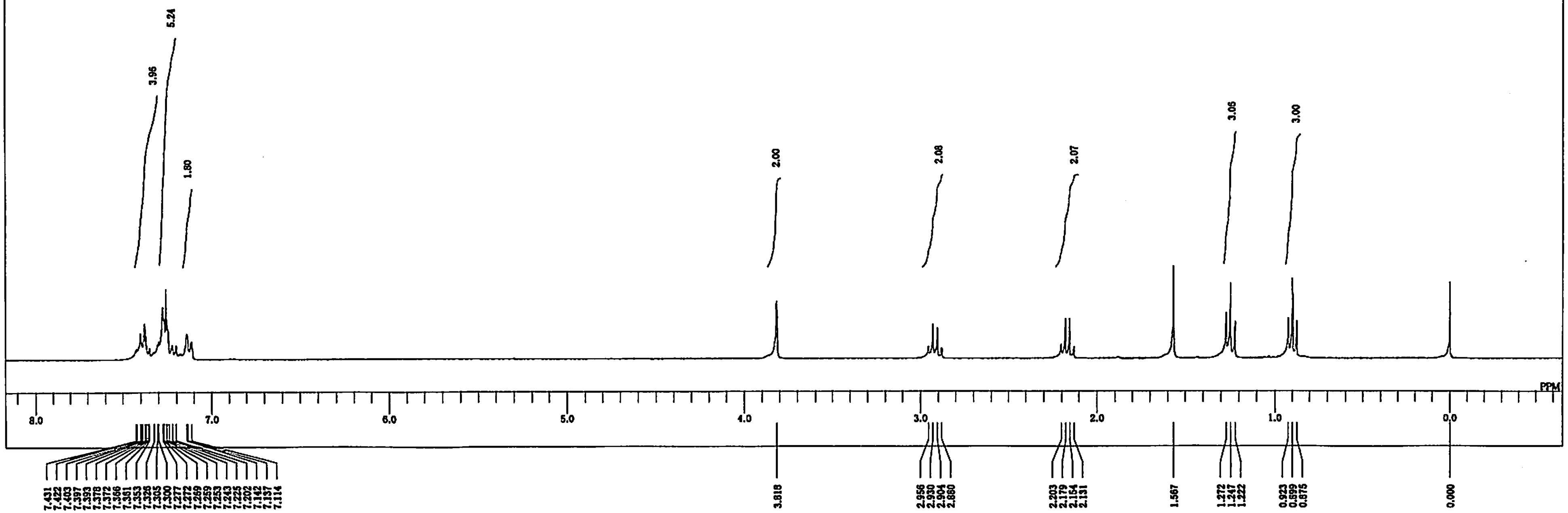

20161025_NO74

¥¥192.168.226.211¥anal¥5年生¥内田¥20161025_NO741BCM_E55.als

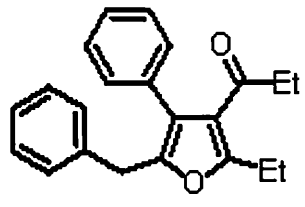

3ab

DFILE 20161025_NO741BCM_E55.als
COMNT 20161025_NO74
DATIM Tue Oct 25 13:08:12 2016
OBNUC 13C
EXMOD BCM
OBFRQ 75.45 MHz
OBSET 124.00 KHz
OBFIN 1840.00 Hz
POINT 32768
FREQU 20356.23 Hz
SCANS 2400
ACQTM 1.6097 sec
PD 1.3900 sec
PW1 5.10 usec
IRNUC 1H
CTEMP 20.4 c
SLVNT CDCL3
EXREF 77.00 ppm
BF 0.12 Hz
RGAIN 26

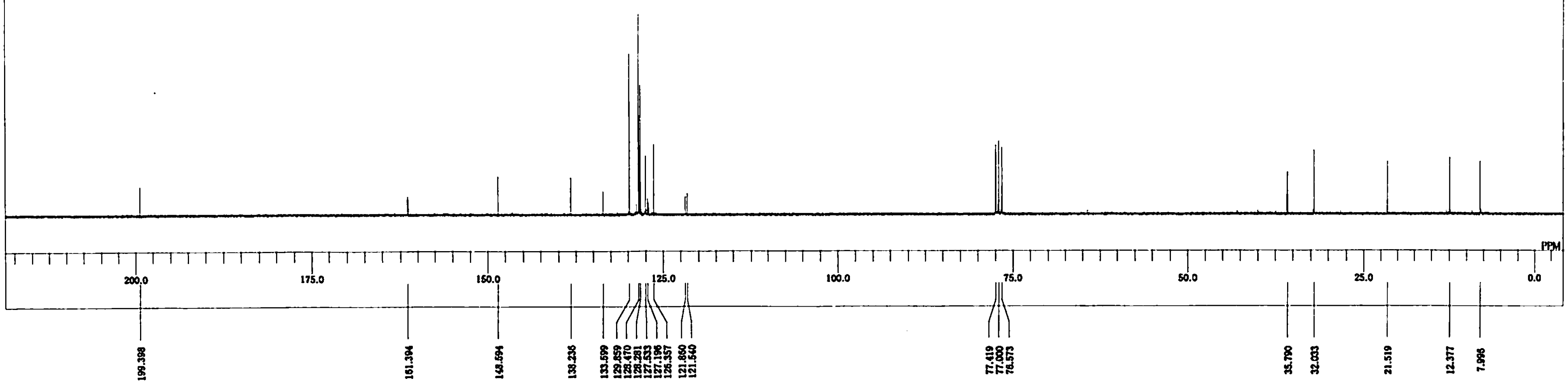

20161129_NO57

¥¥192.168.226.211¥analV5¥[illegible]¥20161129_NO571NON_1126.als

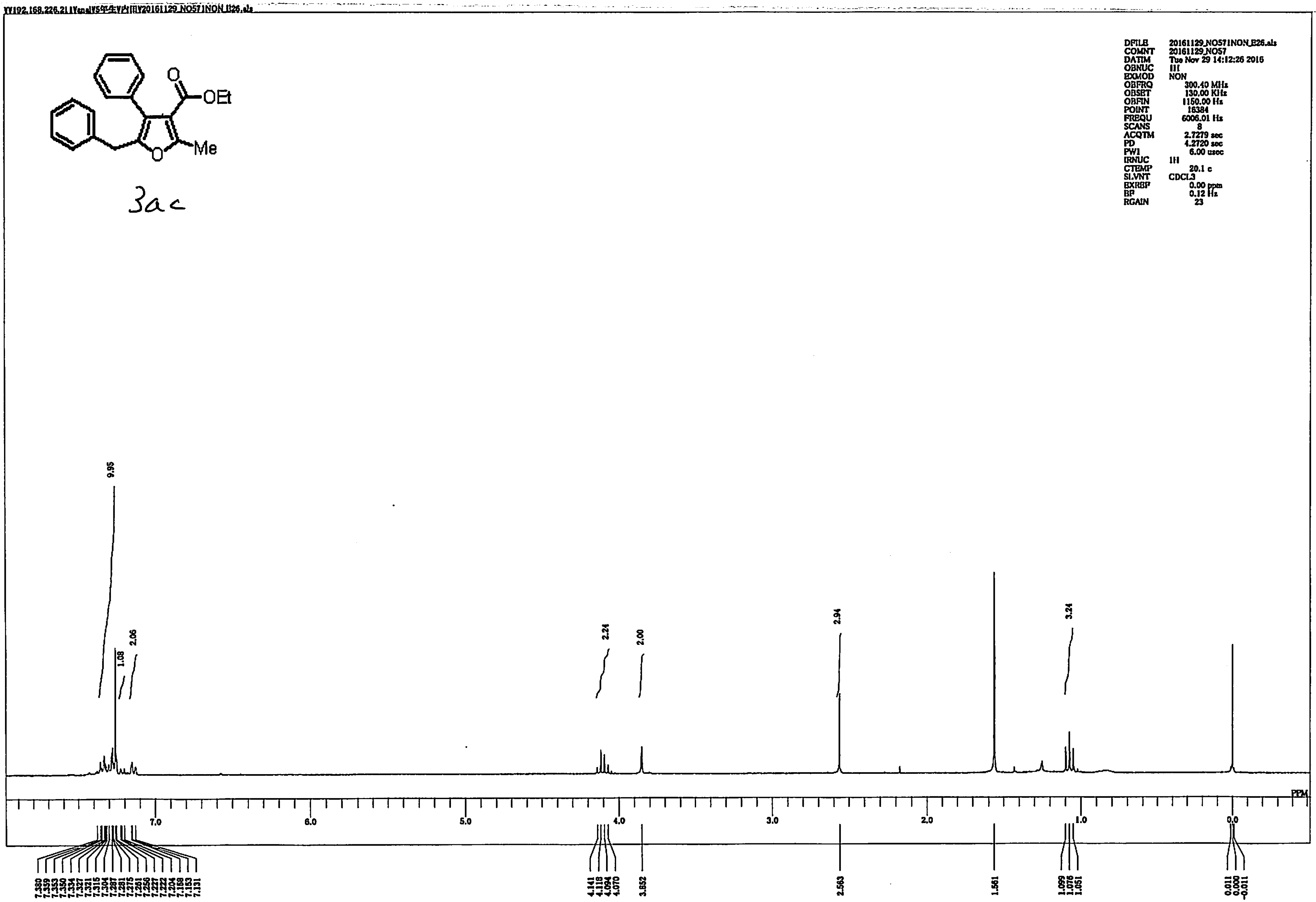

20161212_No57

¥¥192.168.226.211¥anal¥5年生¥内田¥20161212_No571BCM_H1.als

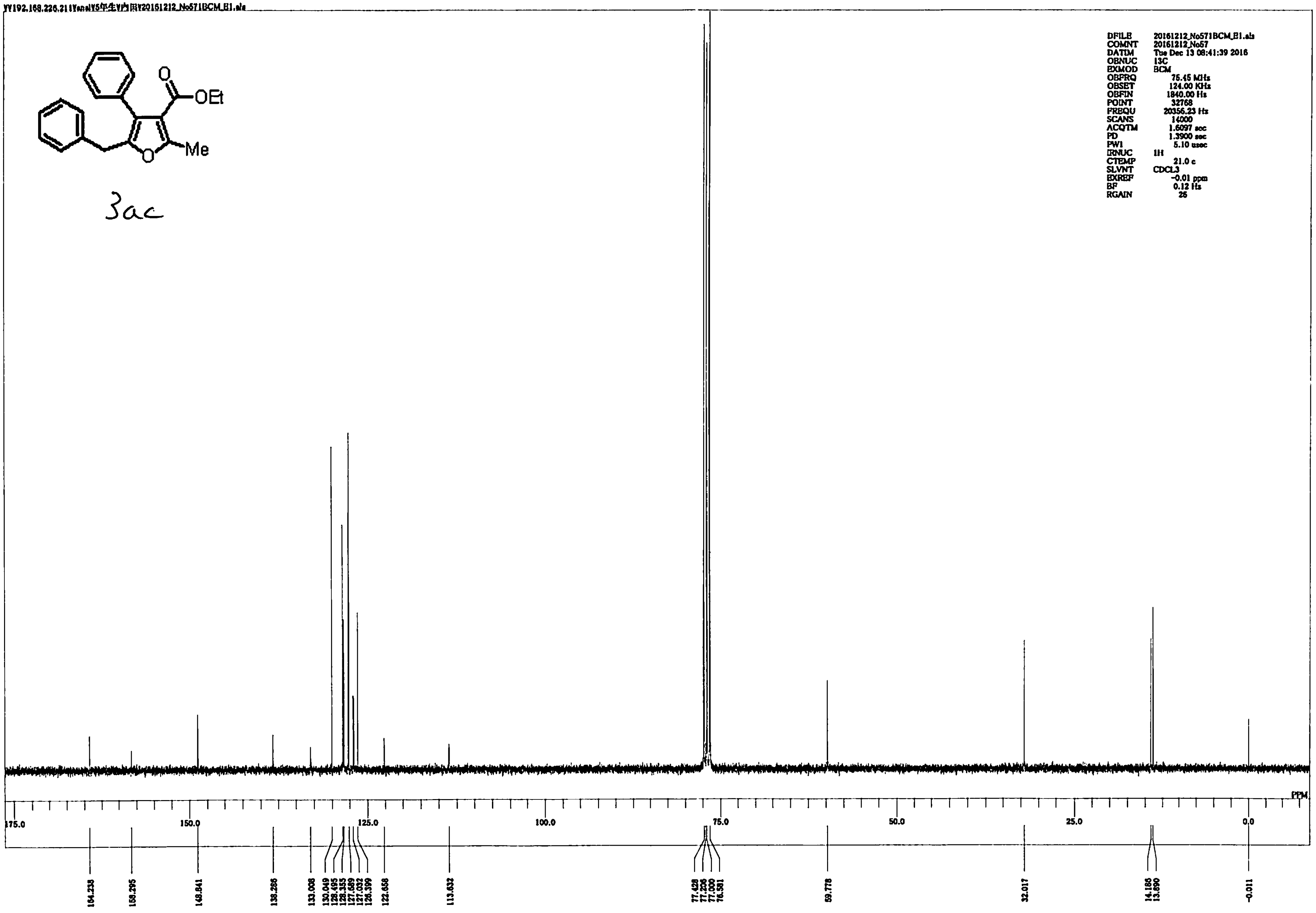

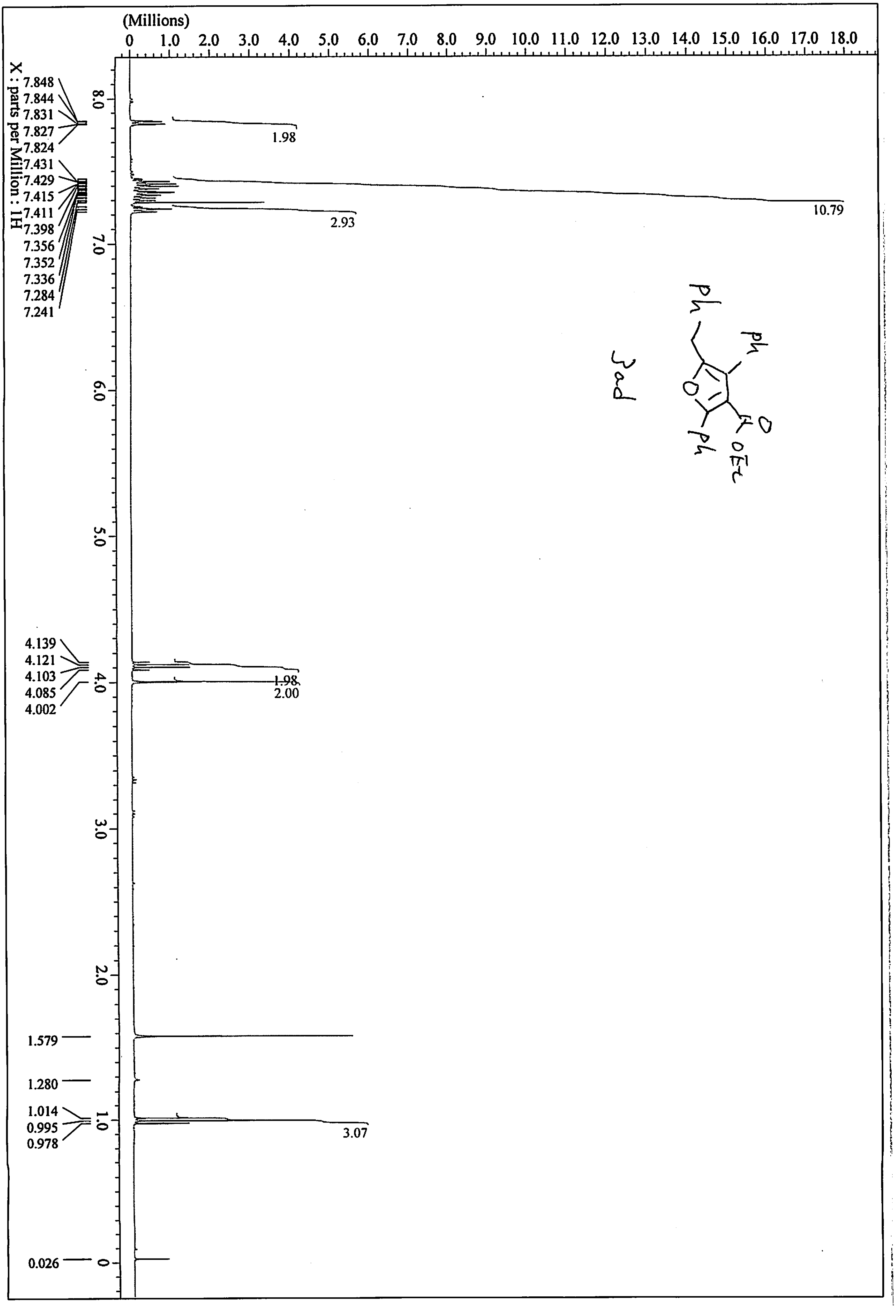
(Millions)
0 1.0 2.0 3.0 4.0 5.0 6.0 7.0 8.0 9.0 10.0 11.0 12.0 13.0 14.0 15.0 16.0 17.0 18.0
X : parts per Million : 1H
7.848
7.844
7.831
7.827
7.824
7.431
7.429
7.415
7.411
7.398
7.356
7.352
7.336
7.284
7.241
4.139
4.121
4.103
4.085
4.002
1.579
1.280
1.014
0.995
0.978
0.026
8.0
7.0
6.0
5.0
4.0
3.0
2.0
1.0
0
1.98
10.79
2.93
1.98
2.00
3.07
3ad
Ph
Ph
Ph
O
OEt

20161217_No78

¥¥192.168.226.211¥and¥5年生¥内田¥20161217_No781BCM_E13.als

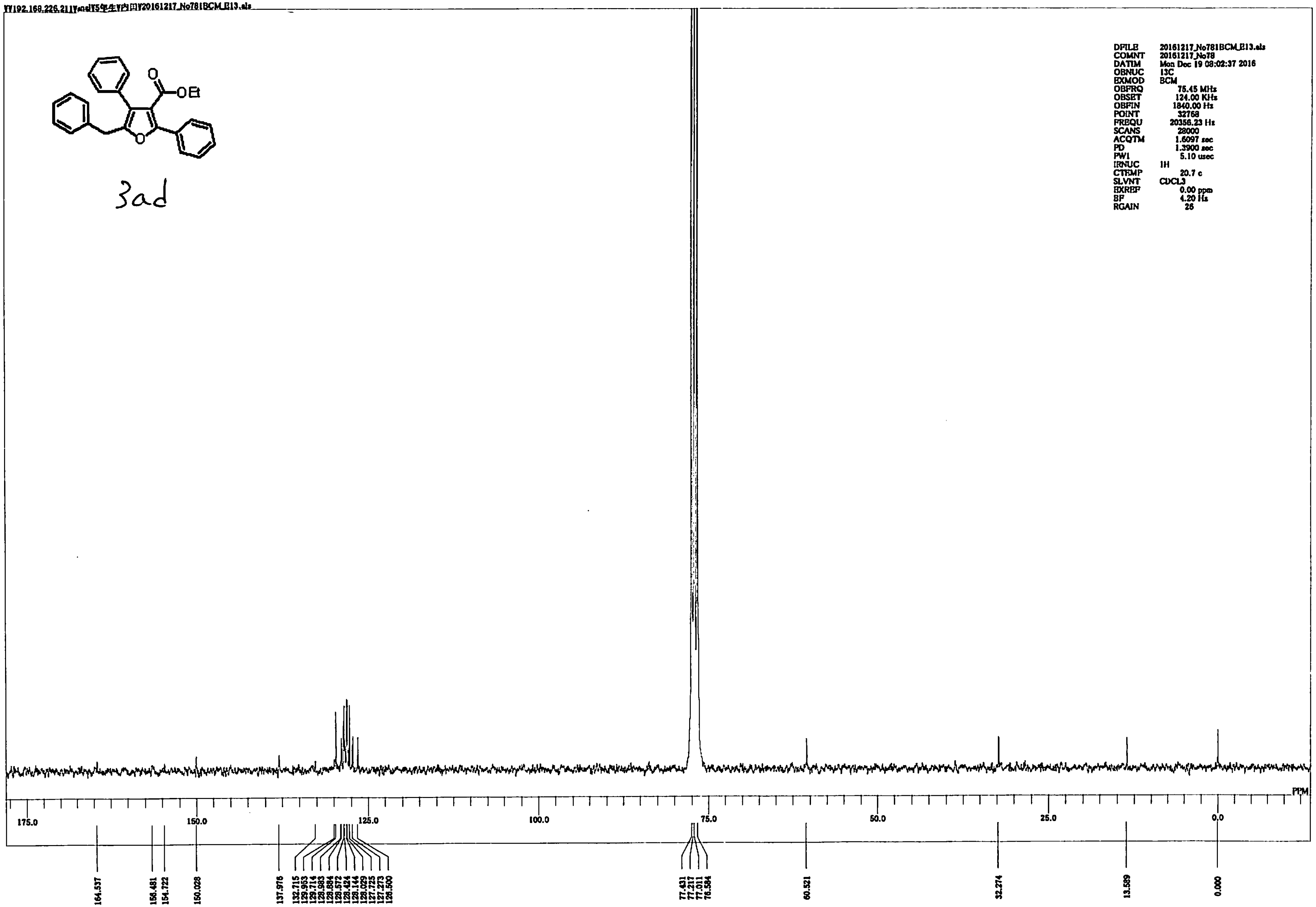

20161122_NO77

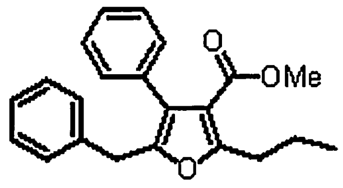

3ae

DFILE	20161122_NO771NON_H12.als
COMNT	20161122_NO77
DATIM	Tue Nov 22 14:13:17 2016
OBNUC	1H
EXMOD	NON
OBFRQ	300.40 MHz
OBSET	130.00 KHz
OBFIN	1150.00 Hz
POINT	16384
FREQU	6006.01 Hz
SCANS	8
ACQTM	2.7279 sec
PD	4.2720 sec
PW1	6.00 usec
IRNUC	1H
CTEMP	20.4 c
SLVNT	CDCL3
EXREF	0.00 ppm
BF	0.12 Hz
RGAIN	24

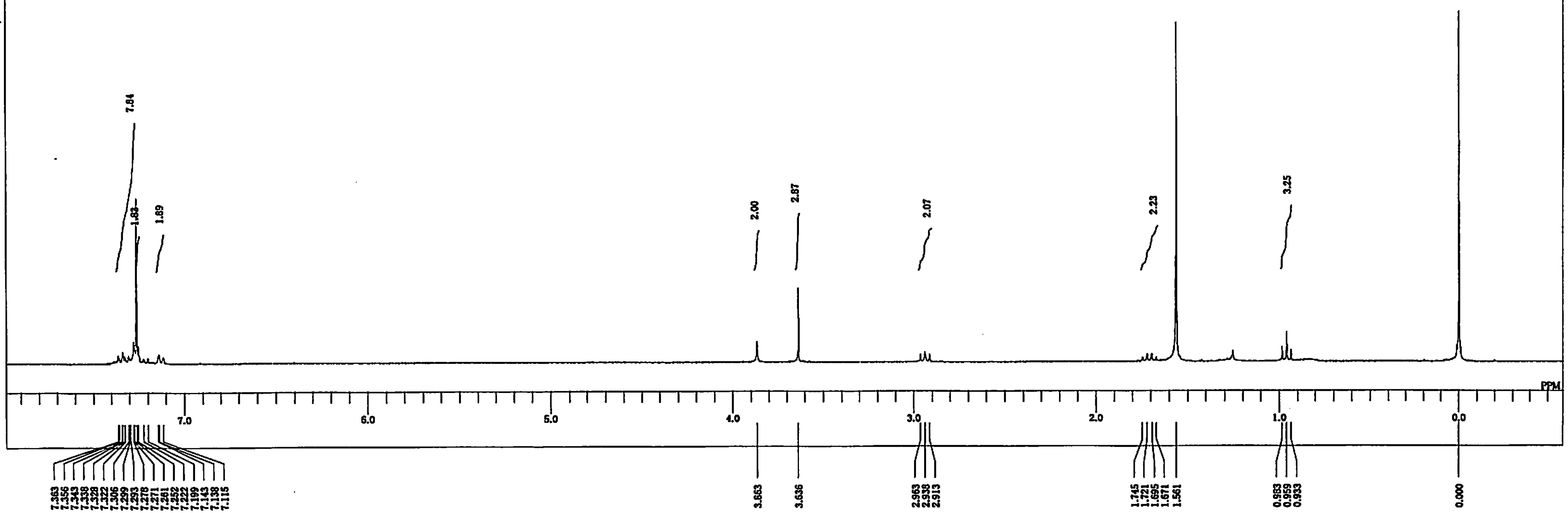

20161202_NO77

\\192.168.226.211\[illegible]\20161202_NO771BCM_B103.als

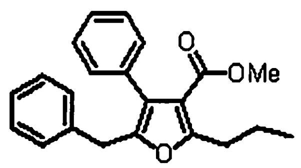

3ae

DFILE 20161202_NO771BCM_B103.als
COMNT 20161202_NO77
DATIM Sat Dec 03 08:43:34 2016
OBNUC 13C
EXMOD BCM
OBFRQ 75.45 MHz
OBSET 124.00 KHz
OBFIN 1840.00 Hz
POINT 32768
FREQU 20356.23 Hz
SCANS 14000
ACQTM 1.6097 sec
PD 1.3900 sec
PW1 5.10 usec
IRNUC 1H
CTEMP 20.8 c
SLVNT CDCL3
EXREF 0.00 ppm
BF 0.12 Hz
RGAIN 26

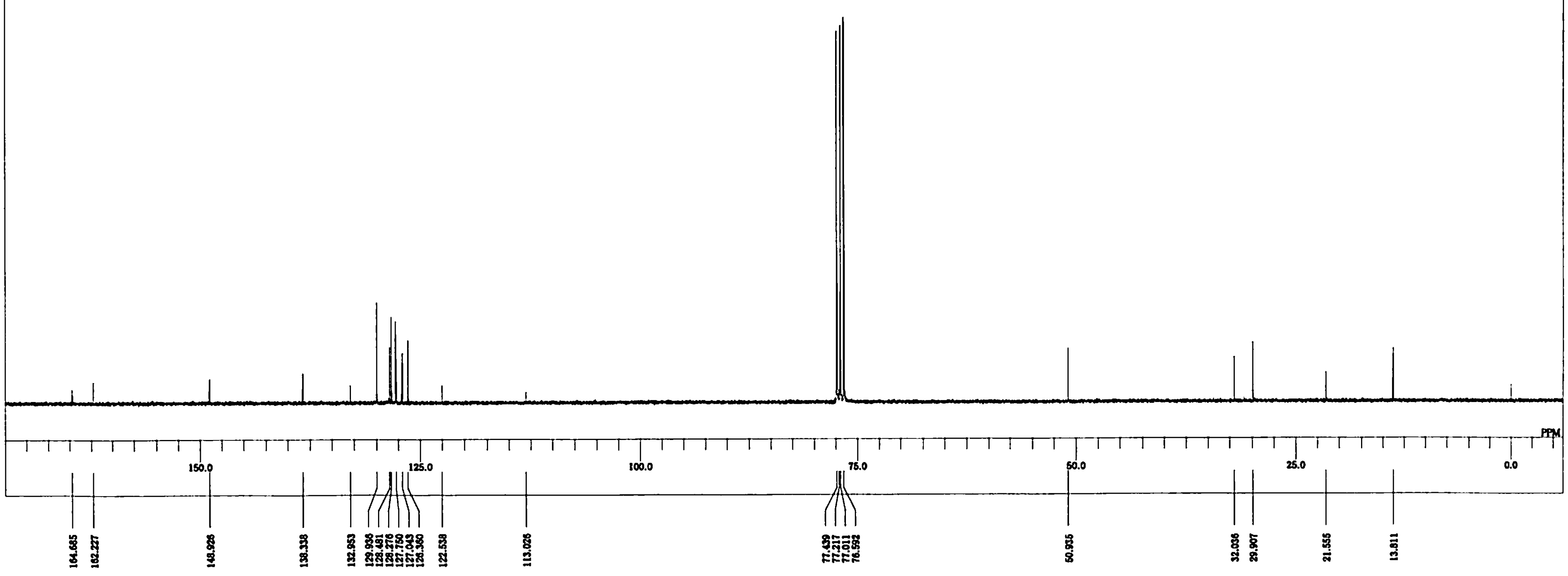

20161017_NO62

¥¥192.168.226.211¥anal¥5年生¥内田¥20161017_NO621NON_E33.als

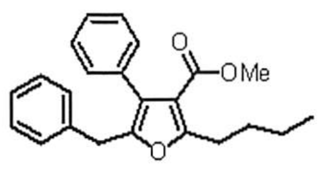

3af

DFILE	20161017_NO621NON_E33.als
COMNT	20161017_NO62
DATIM	Mon Oct 17 13:59:00 2016
OBNUC	1H
EXMOD	NON
OBFRQ	300.40 MHz
OBSET	130.00 KHz
OBFIN	1150.00 Hz
POINT	16384
FREQU	6006.01 Hz
SCANS	8
ACQTM	2.7279 sec
PD	4.2720 sec
PW1	6.00 usec
IRNUC	1H
CTEMP	20.2 c
SLVNT	CDCL3
EXREF	0.00 ppm
BF	0.12 Hz
RGAIN	14

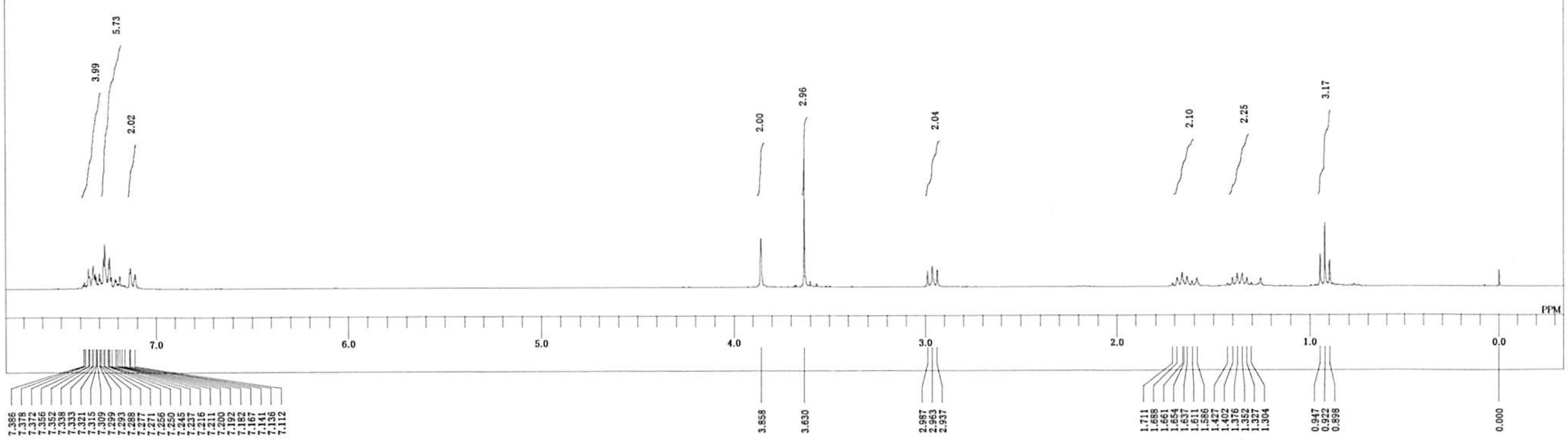

20161130_No62

¥¥192.168.226.211¥anal¥5年生¥内田¥分子内環化反応¥20161130_No621BCM_B58.als

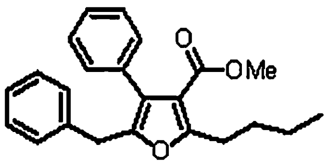

3af

DFILE	20161130_No621BCM_B58.als
COMNT	20161130_No62
DATIM	Thu Dec 01 08:37:59 2016
OBNUC	13C
EXMOD	BCM
OBFRQ	75.45 MHz
OBSET	124.00 KHz
OBFIN	1840.00 Hz
POINT	32768
FREQU	20356.23 Hz
SCANS	14000
ACQTM	1.6097 sec
PD	1.3900 sec
PW1	5.10 usec
IRNUC	1H
CTEMP	21.5 c
SLVNT	CDCL3
EXREF	77.00 ppm
BF	0.12 Hz
RGAIN	26

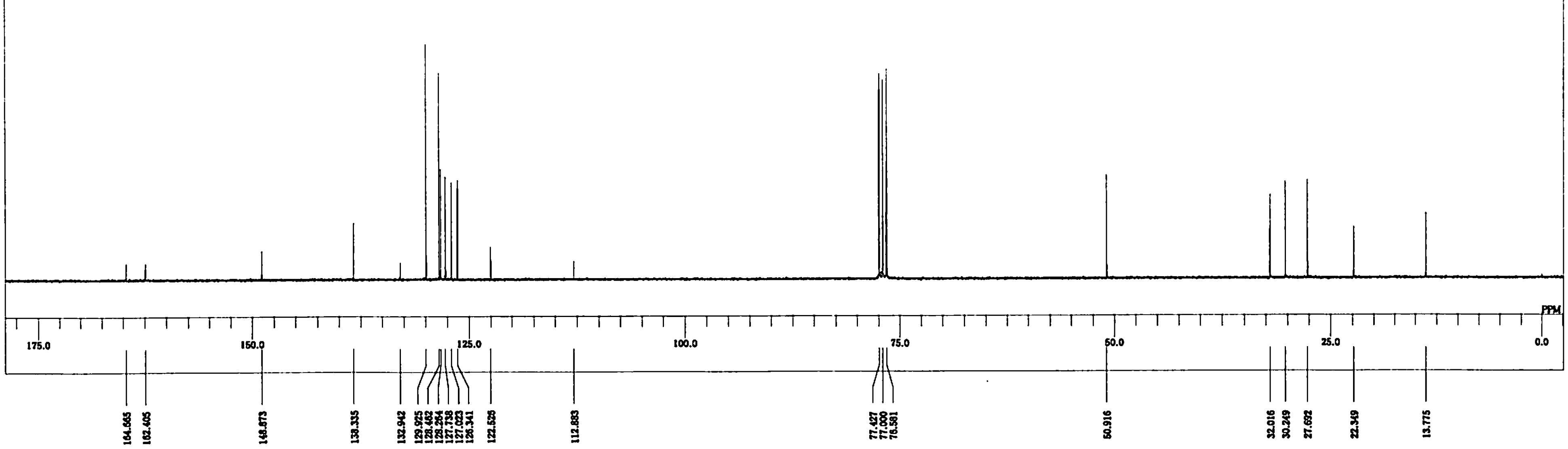

20160915_NO63_5to7

D:¥data¥5ﾃ?ｨ¥ﾌﾟ¥[illegible]¥20160915_NO63_5to71NON_H1.als

DFILE	20160915_NO63_5to71NON_H1.als
COMNT	20160915_NO63_5to7
DATIM	Thu Sep 15 12:46:13 2016
OBNUC	1H
EXMOD	NON
OBFRQ	300.40 MHz
OBSET	130.00 KHz
OBFIN	1150.00 Hz
POINT	16384
FREQU	6006.01 Hz
SCANS	16
ACQTM	2.7279 sec
PD	4.2720 sec
PW1	6.00 usec
IRNUC	1H
CTEMP	21.8 c
SLVNT	CDCL3
EXREF	0.00 ppm
BF	0.12 Hz
RGAIN	19

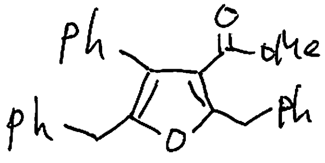

3ag

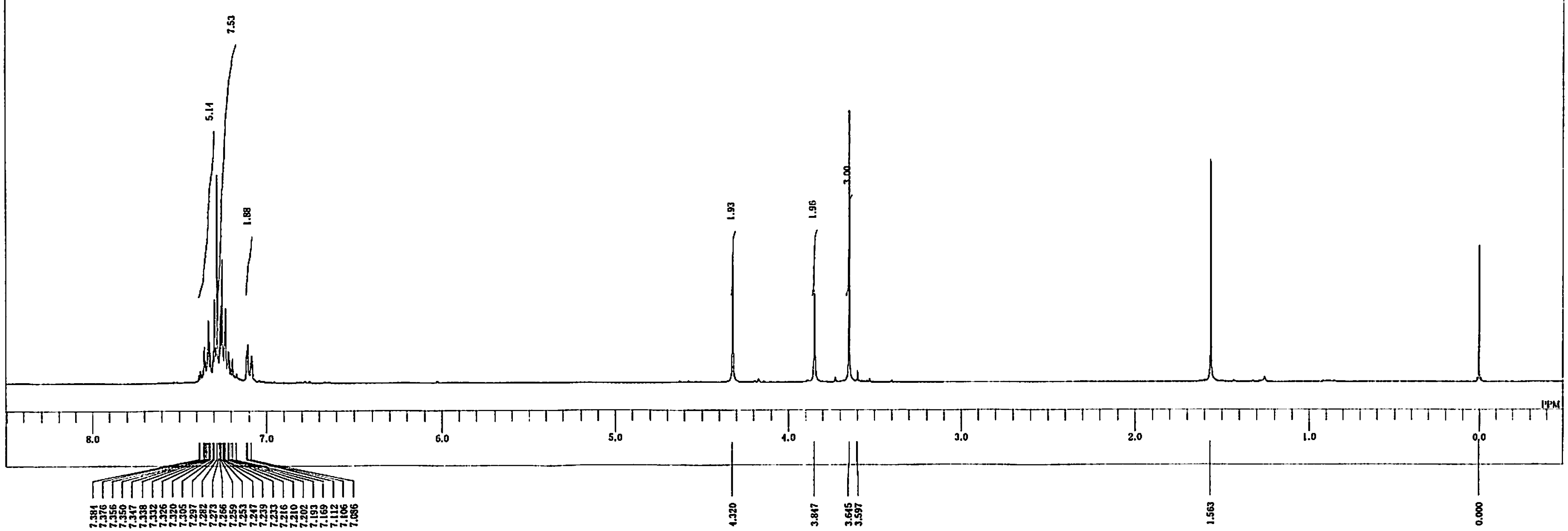

20161208_No63

¥¥192.168.226.211¥enal¥5中生¥内田¥20161208_No632BCM_B50.als

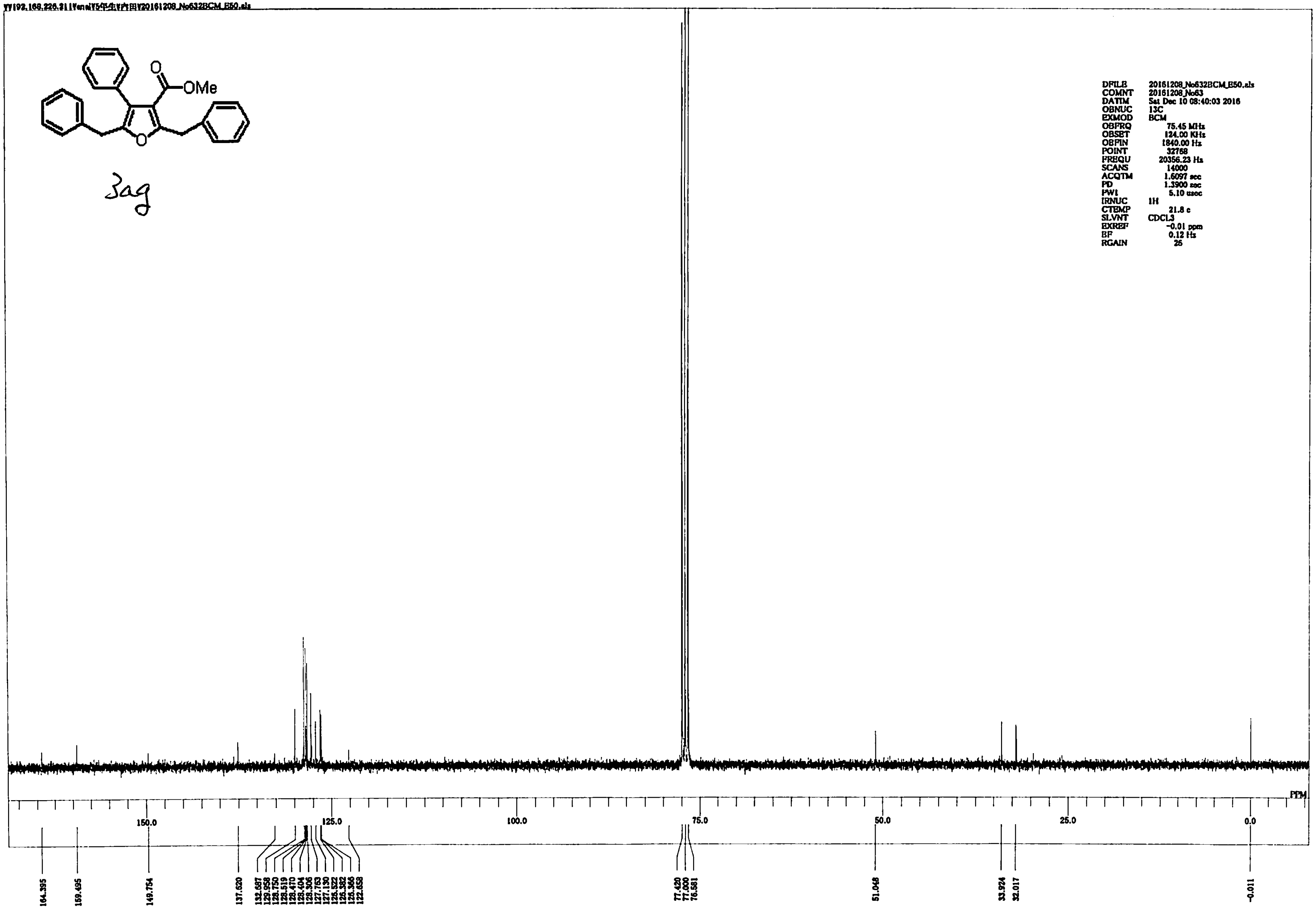

161201NM518

\\192.168.226.211\[illegible]\161201NM5181NON_B59.als

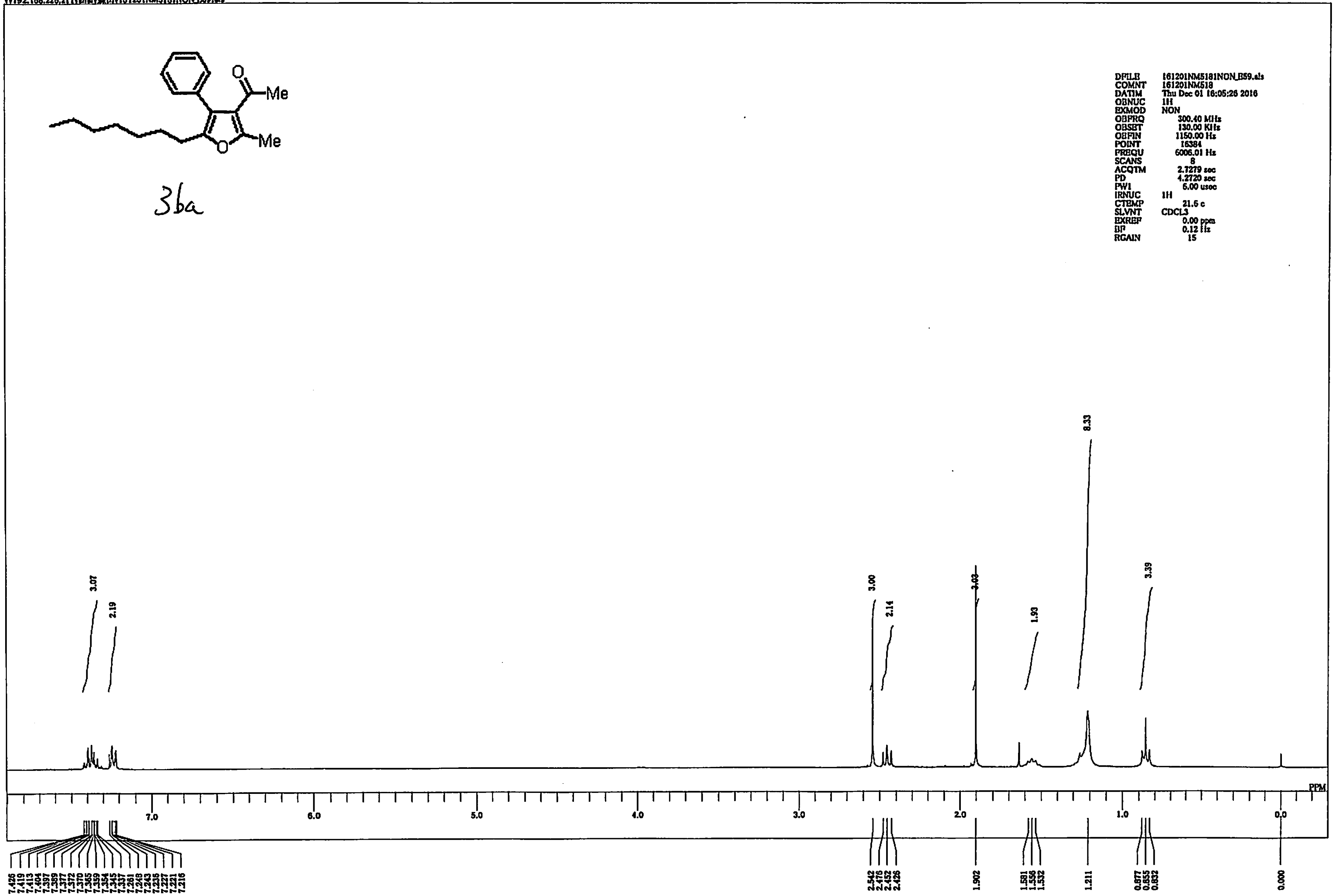

161201NM518

¥¥192.168.226.211¥anal¥受託¥161201NM5181BCM_E67.als

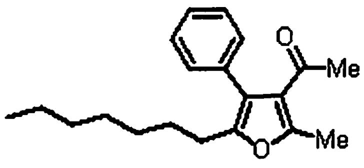

3ba

DFILE	161201NM5181BCM_E67.als
COMNT	161201NM518
DATIM	Fri Dec 02 01:05:15 2016
OBNUC	13C
EXMOD	BCM
OBFRQ	75.45 MHz
OBSET	124.00 KHz
OBFIN	1840.00 Hz
POINT	32768
FREQU	20356.23 Hz
SCANS	6000
ACQTM	1.6097 sec
PD	1.3900 sec
PW1	5.10 usec
IRNUC	1H
CTEMP	20.9 c
SLVNT	CDCL3
EXREF	77.00 ppm
BF	0.12 Hz
RGAIN	25

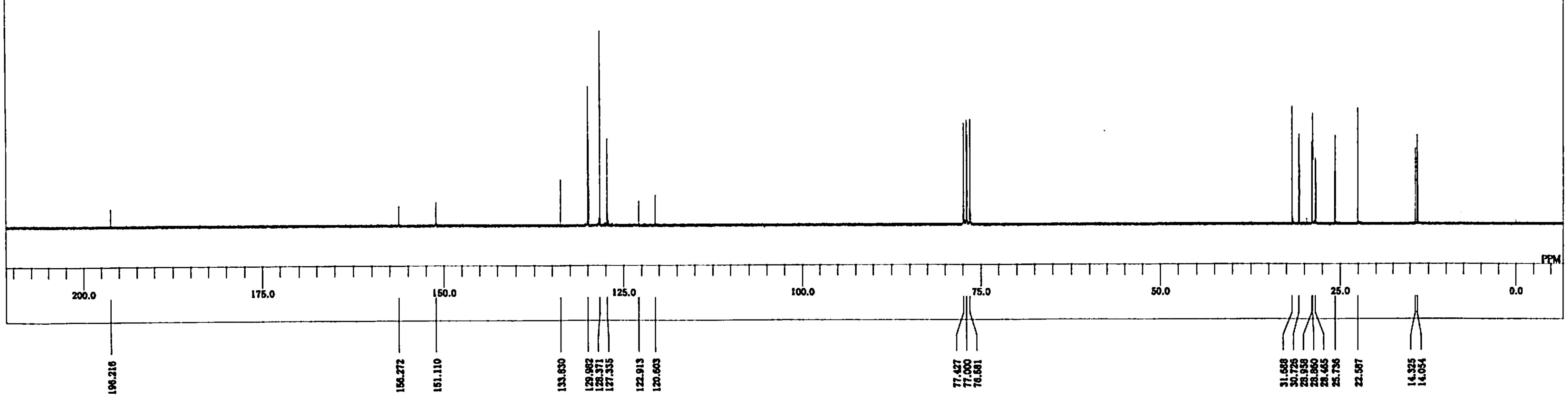

161206NM520_new_1

\\192.168.226.21\[illegible]\161206NM520_new_11NON_B17.als

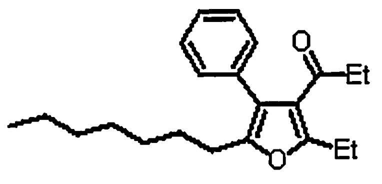

3bb

DFILE	161206NM520_new_11NON_B17.als
COMNT	161206NM520_new_1
DATIM	Tue Dec 06 19:05:50 2016
OBNUC	1H
EXMOD	NON
OBFRQ	300.40 MHz
OBSET	130.00 KHz
OBFIN	1150.00 Hz
POINT	16384
FREQU	6006.01 Hz
SCANS	8
ACQTM	2.7279 sec
PD	4.2720 sec
PW1	6.00 usec
IRNUC	1H
CTEMP	20.5 c
SLVNT	CDCL3
EXREF	0.00 ppm
BF	0.12 Hz
RGAIN	16

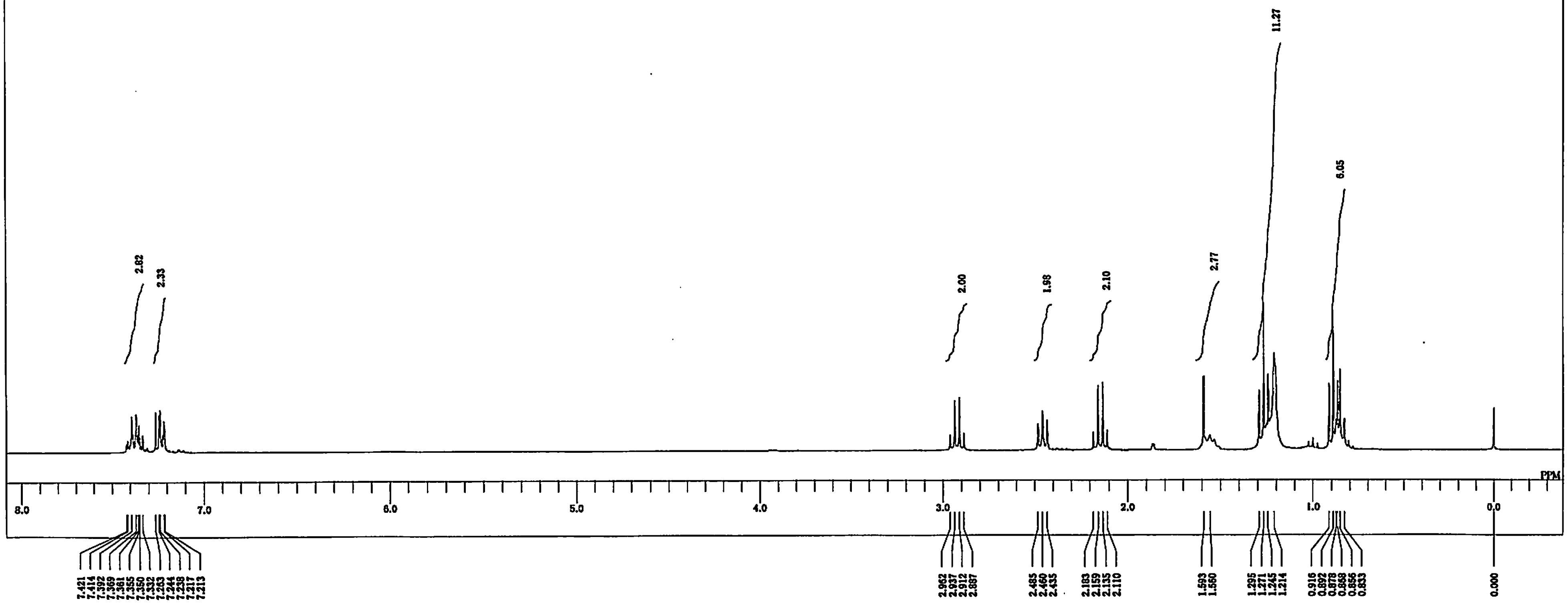

161220NM520
¥¥192.168.226.211¥[illegible]¥161220NM5201BCM_E2.als

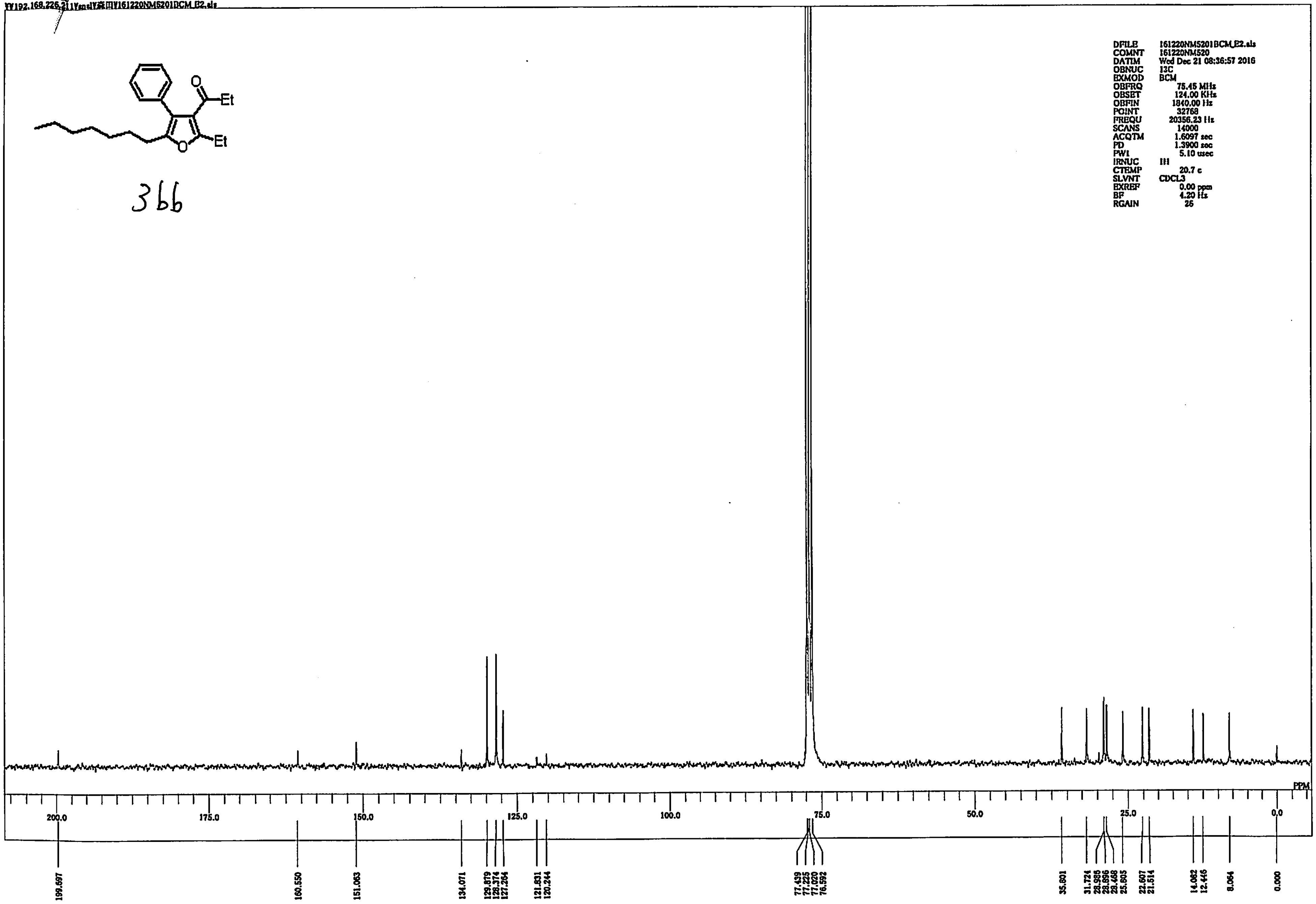

161203NM521_1

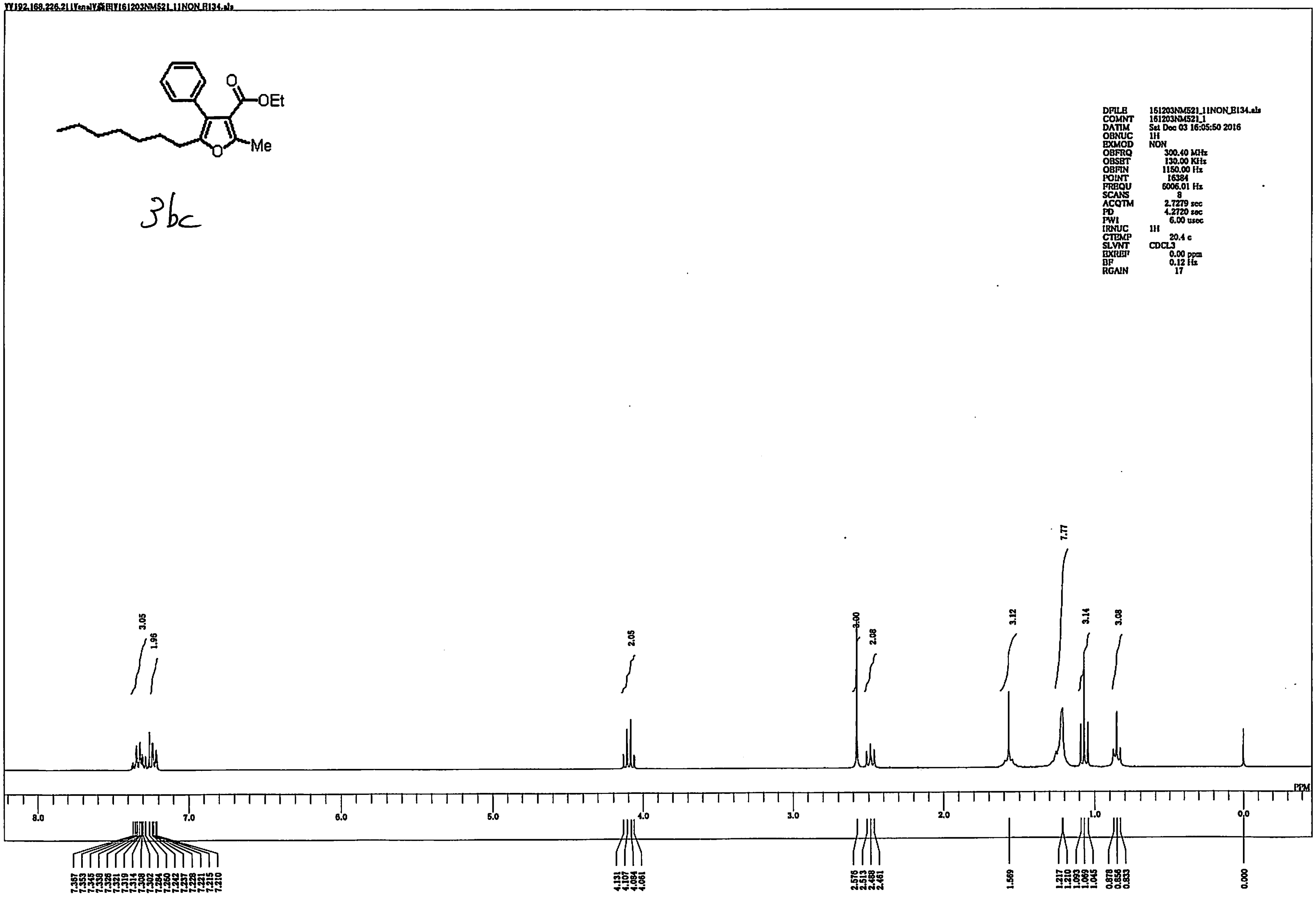

161203NM521_1

¥¥192.168.226.21¥[illegible]¥161203NM521_11BCM_B137.als

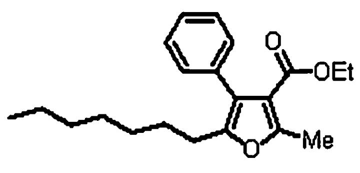

3bc

DFILE	161203NM521_11BCM_B137.als
COMNT	161203NM521_1
DATIM	Sun Dec 04 08:31:55 2016
OBNUC	13C
EXMOD	BCM
OBFRQ	75.45 MHz
OBSET	124.00 KHz
OBFIN	1840.00 Hz
POINT	32768
FREQU	20356.23 Hz
SCANS	14000
ACQTM	1.6097 sec
PD	1.3900 sec
PW1	5.10 usec
IRNUC	1H
CTEMP	20.8 c
SLVNT	CDCL3
EXREF	77.00 ppm
BF	0.12 Hz
RGAIN	25

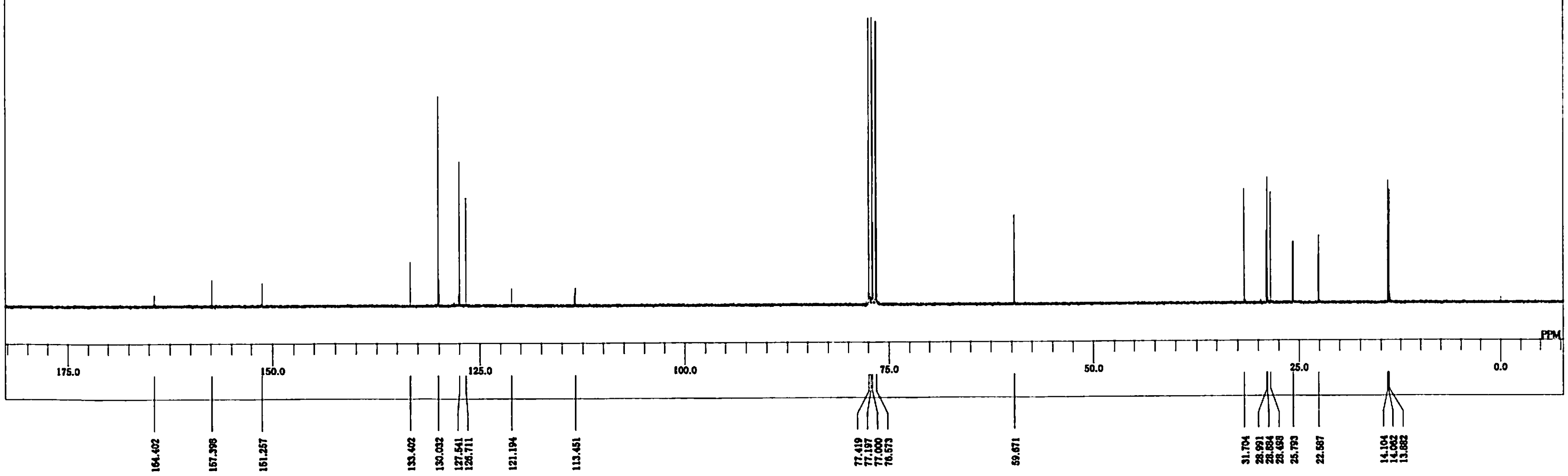

161215NM523_2

\\192.168.226.21\Venal\YKIII\161215NM523_21NON_E8.als

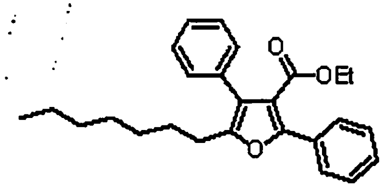

3bd

DFILE	161215NM523_21NON_E8.als
COMNT	161215NM523_2
DATIM	Thu Dec 15 13:55:22 2016
OBNUC	1H
EXMOD	NON
OBFRQ	300.40 MHz
OBSET	130.00 KHz
OBFIN	1150.00 Hz
POINT	16384
FREQU	6006.01 Hz
SCANS	8
ACQTM	2.7279 sec
PD	4.2720 sec
PW1	6.00 usec
IRNUC	1H
CTEMP	19.7 c
SLVNT	CDCL3
EXREF	0.00 ppm
BF	0.12 Hz
RGAIN	15

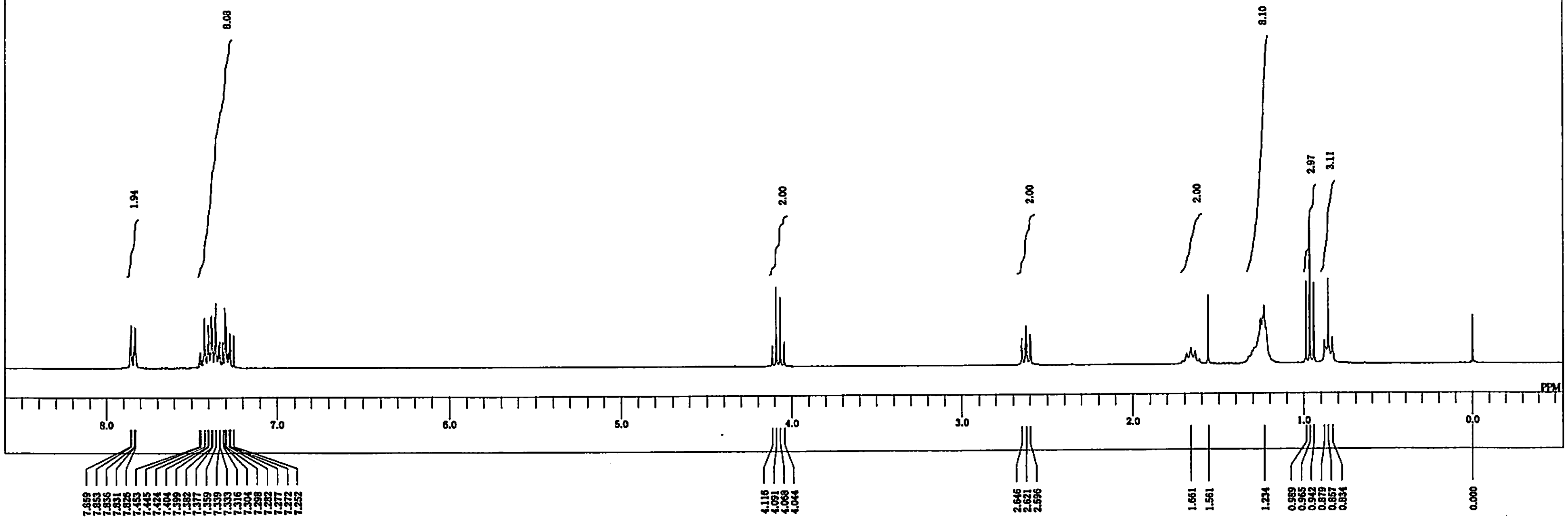

161215NM523_2

\\192.168.226.211\\[illegible]\\161215NM523_21BCM_B18.als

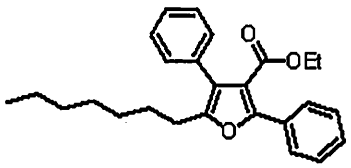

3bd

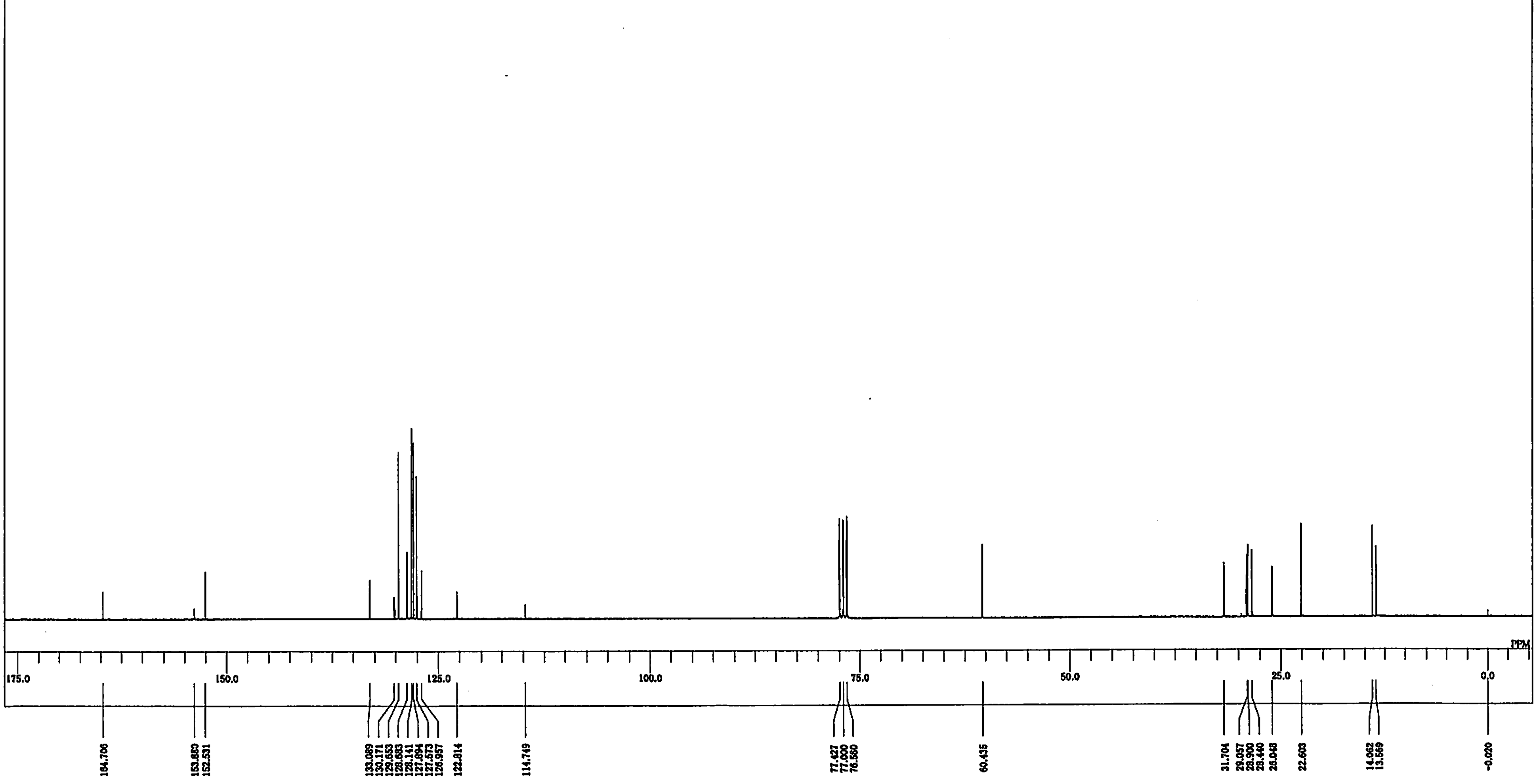

161217NM524_1

YY192.168.226.211¥enst¥宏田¥161217NM524-11NON.B1.als

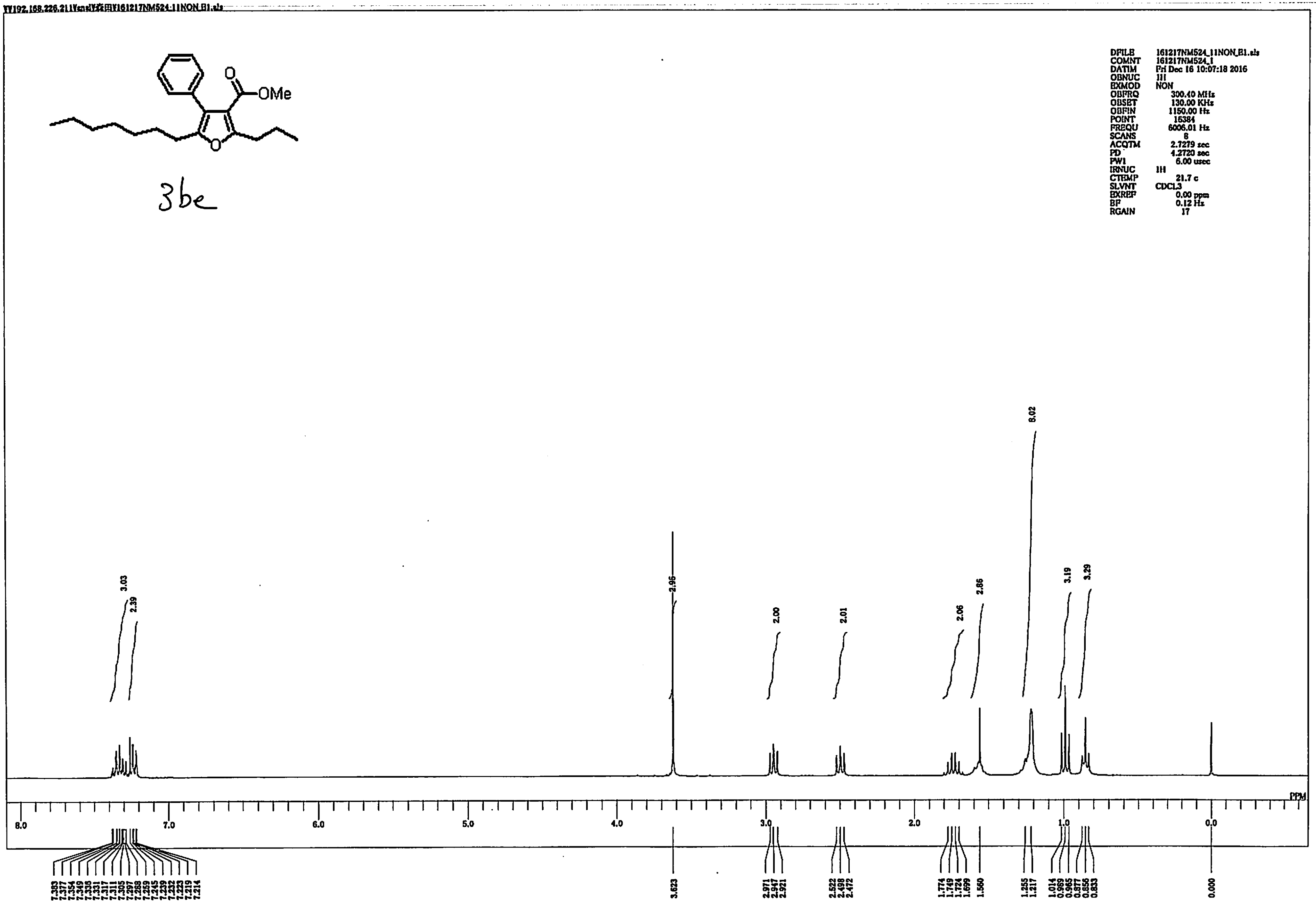

161217NM524_1

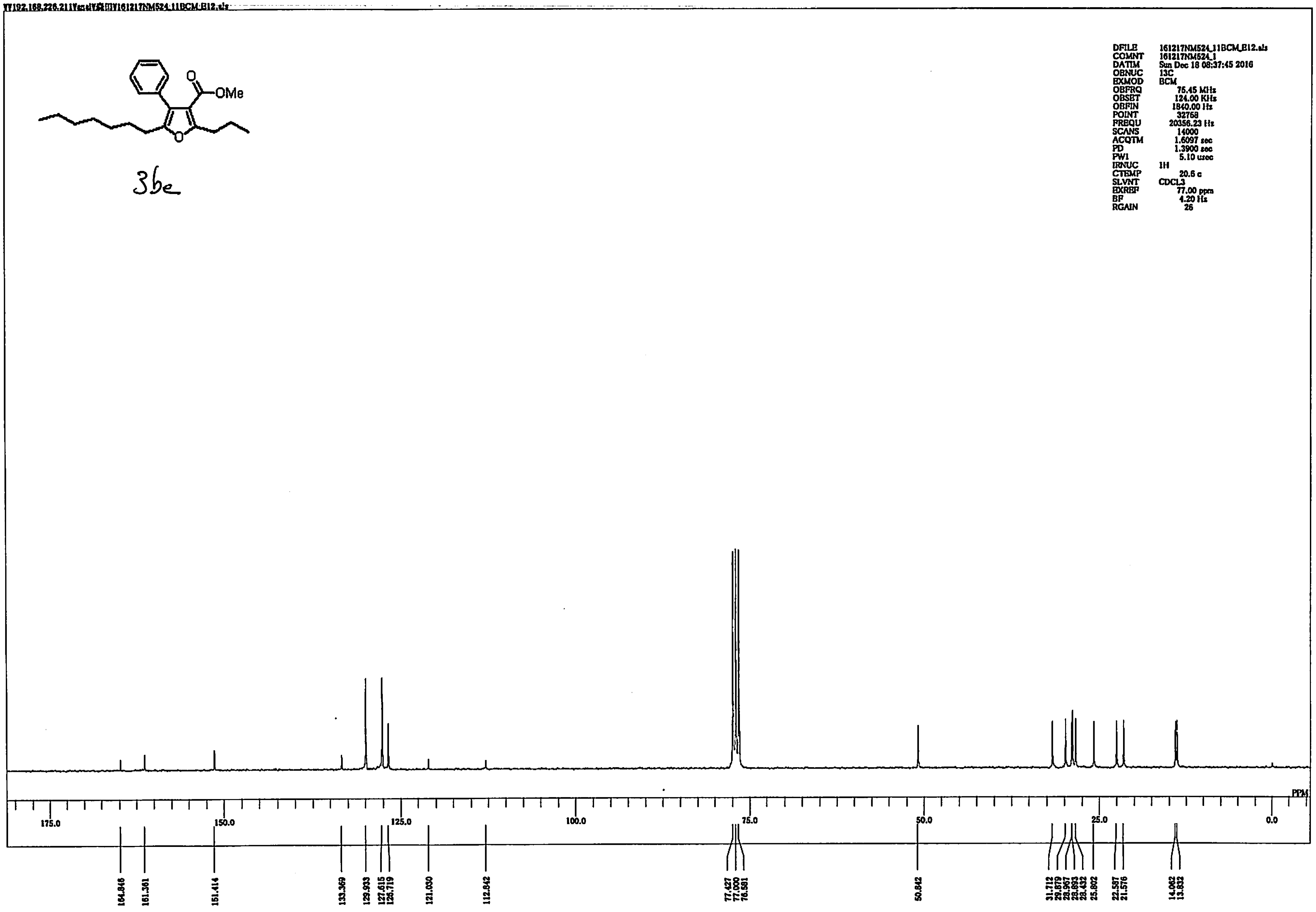

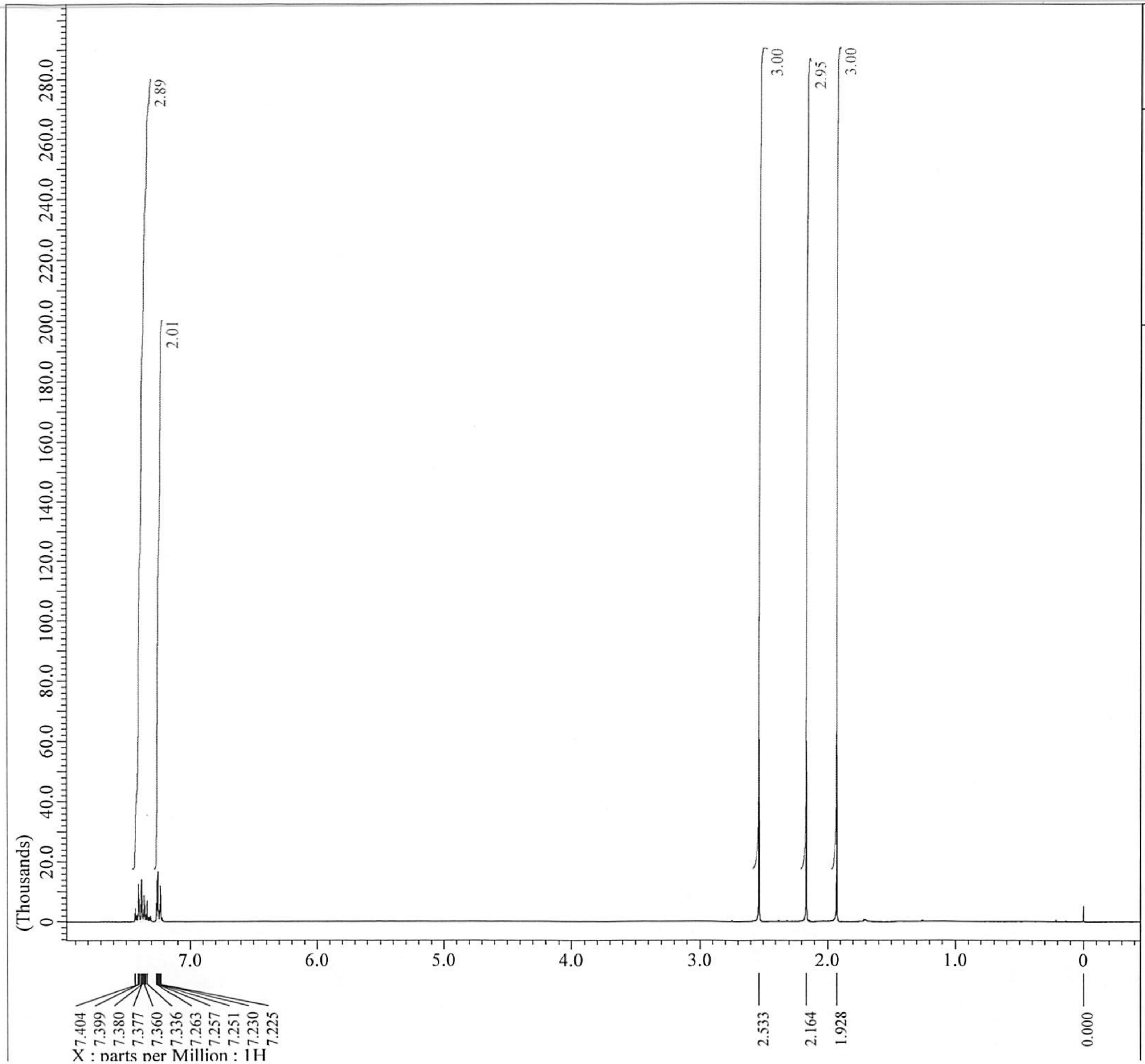

JEOL
Solutions for Innovation

---- PROCESSING PARAMETERS ----
dc_balance(0, FALSE)
sexp(0.2[Hz], 0.0[s])
trapezoid(0[%], 0[%], 80[%], 100[%])
zerofill(1)
fft(1, TRUE, TRUE)
machinephase
ppm

以下に由来:: Morita_3010-1.jdf

Filename	=	Morita_3010-3.jdf
Author	=	Administrator
Experiment	=	zg30
Sample_Id	=	NM1061_Fr7
Solvent	=	CHLOROFORM-D
Creation_Time	=	23-DEC-2022 14:14:19
Revision_Time	=	23-DEC-2022 14:15:47
Current_Time	=	23-DEC-2022 14:15:59
Comment	=	NM1061_Fr7
Data_Format	=	1D COMPLEX
Dim_Size	=	32768
Dim_Title	=	1H
Dim_Units	=	[ppm]
Dimensions	=	X
Spectrometer	=	BRUKER_DMX_NMR
Field_Strength	=	7.0492145[T] (300[MHz])
X_Domain	=	1H
X_Freq	=	300.13185343[MHz]
X_Freq_Flip	=	TRUE
X_Offset	=	1.85342561[kHz]
X_Points	=	32768
X_Prescans	=	2
X_Sweep	=	6.18811881[kHz]
Scans	=	16
Temp_Get	=	294.56[K]
Filter_Factor	=	3232

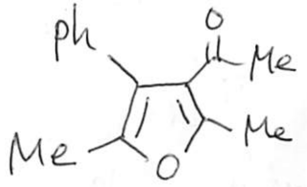

3da

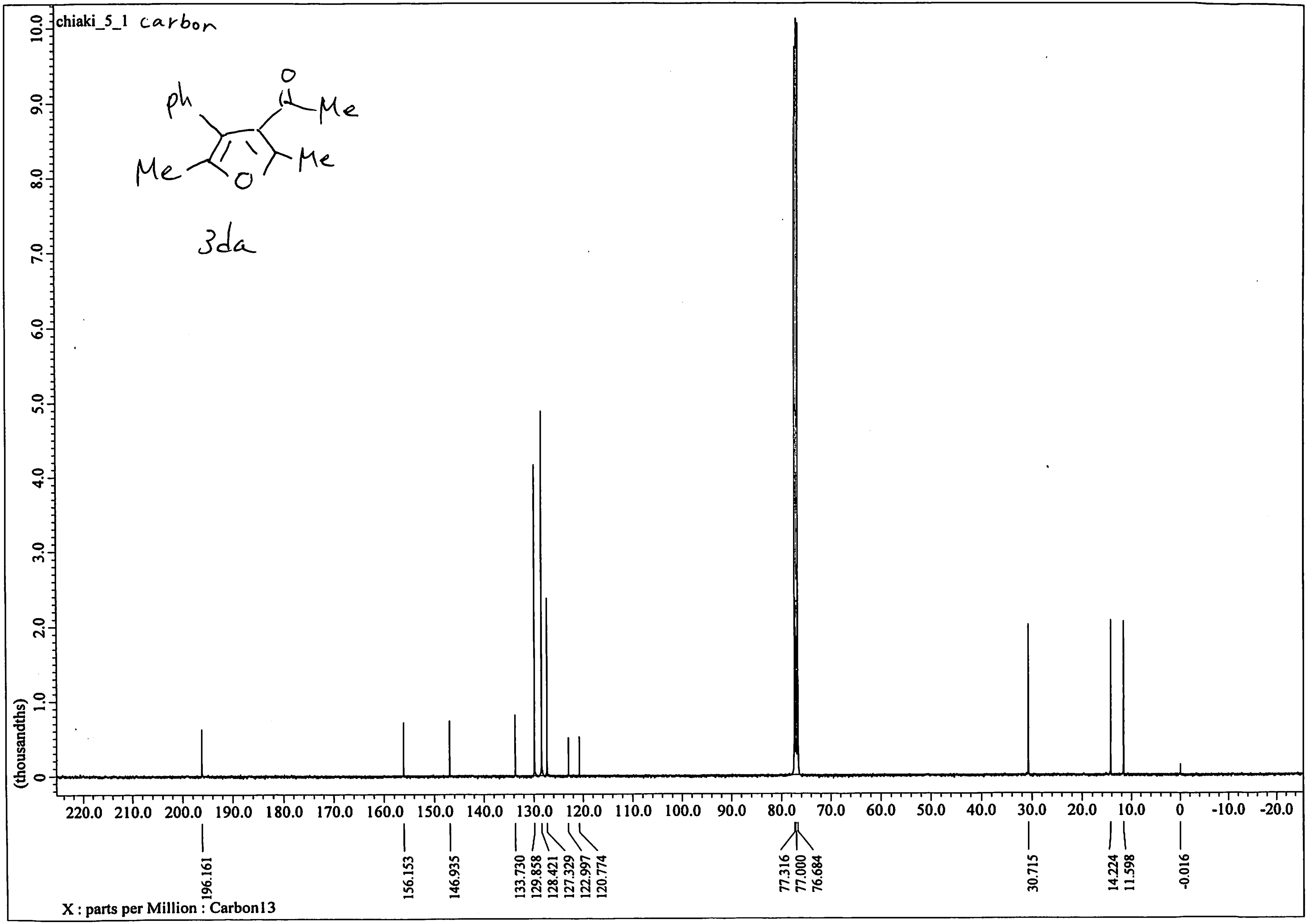
chiaki_5_1 carbon
ph
O
Me
Me
Me
3da
(thousandths)
0
1.0
2.0
3.0
4.0
5.0
6.0
7.0
8.0
9.0
10.0
220.0 210.0 200.0 190.0 180.0 170.0 160.0 150.0 140.0 130.0 120.0 110.0 100.0 90.0 80.0 70.0 60.0 50.0 40.0 30.0 20.0 10.0 0 -10.0 -20.0
196.161
156.153
146.935
133.730
129.858
128.421
127.329
122.997
120.774
77.316
77.000
76.684
30.715
14.224
11.598
-0.016
X : parts per Million : Carbon13

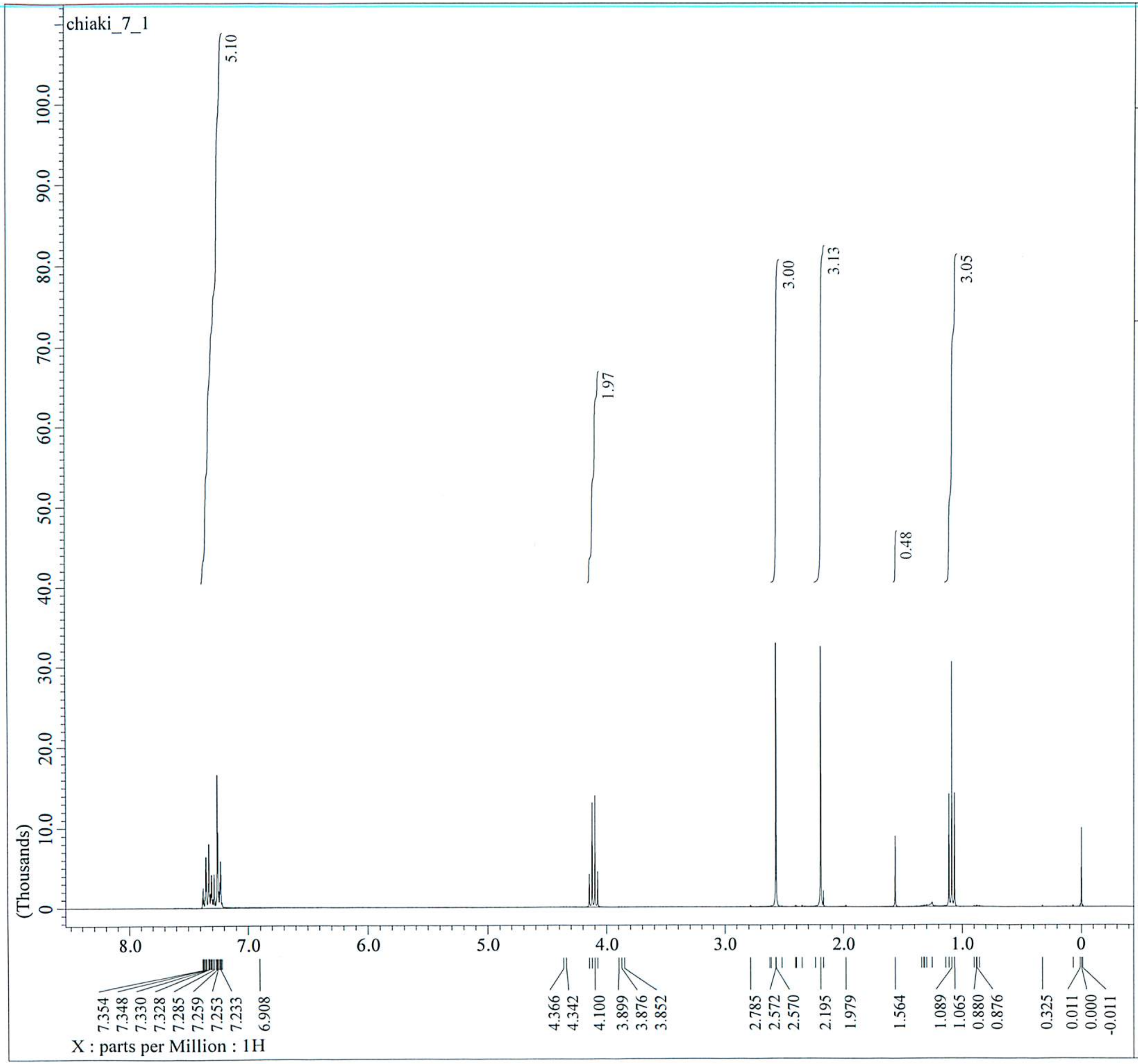

---- PROCESSING PARAMETERS ----
dc_balance(0, FALSE)
sexp(0.2[Hz], 0.0[s])
trapezoid(0[%], 0[%], 80[%], 100[%])
zerofill(1)
fft(1, TRUE, TRUE)
machinephase
ppm

Filename	= Y-18_Kunugi_1620-3.jdf
Author	= Administrator
Experiment	= zg30
Sample_Id	= chiaki_7_1
Solvent	= CHLOROFORM-D
Creation_Time	= 8-FEB-2020 13:45:47
Revision_Time	= 13-FEB-2020 15:17:46
Current_Time	= 13-FEB-2020 15:18:02
Comment	= chiaki_7_1
Data_Format	= 1D COMPLEX
Dim_Size	= 32768
Dim_Title	= 1H
Dim_Units	= [ppm]
Dimensions	= X
Spectrometer	= BRUKER_DMX_NMR
Field_Strength	= 7.0492145[T] (300[MHz])
X_Domain	= 1H
X_Freq	= 300.13185343[MHz]
X_Freq_Flip	= TRUE
X_Offset	= 1.85342561[kHz]
X_Points	= 32768
X_Prescans	= 2
X_Sweep	= 6.18811881[kHz]
Scans	= 16
Temp_Get	= 294.46[K]
Filter_Factor	= 3232

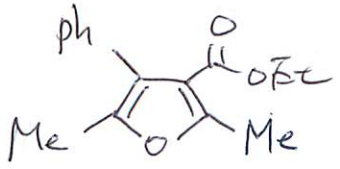

3dd

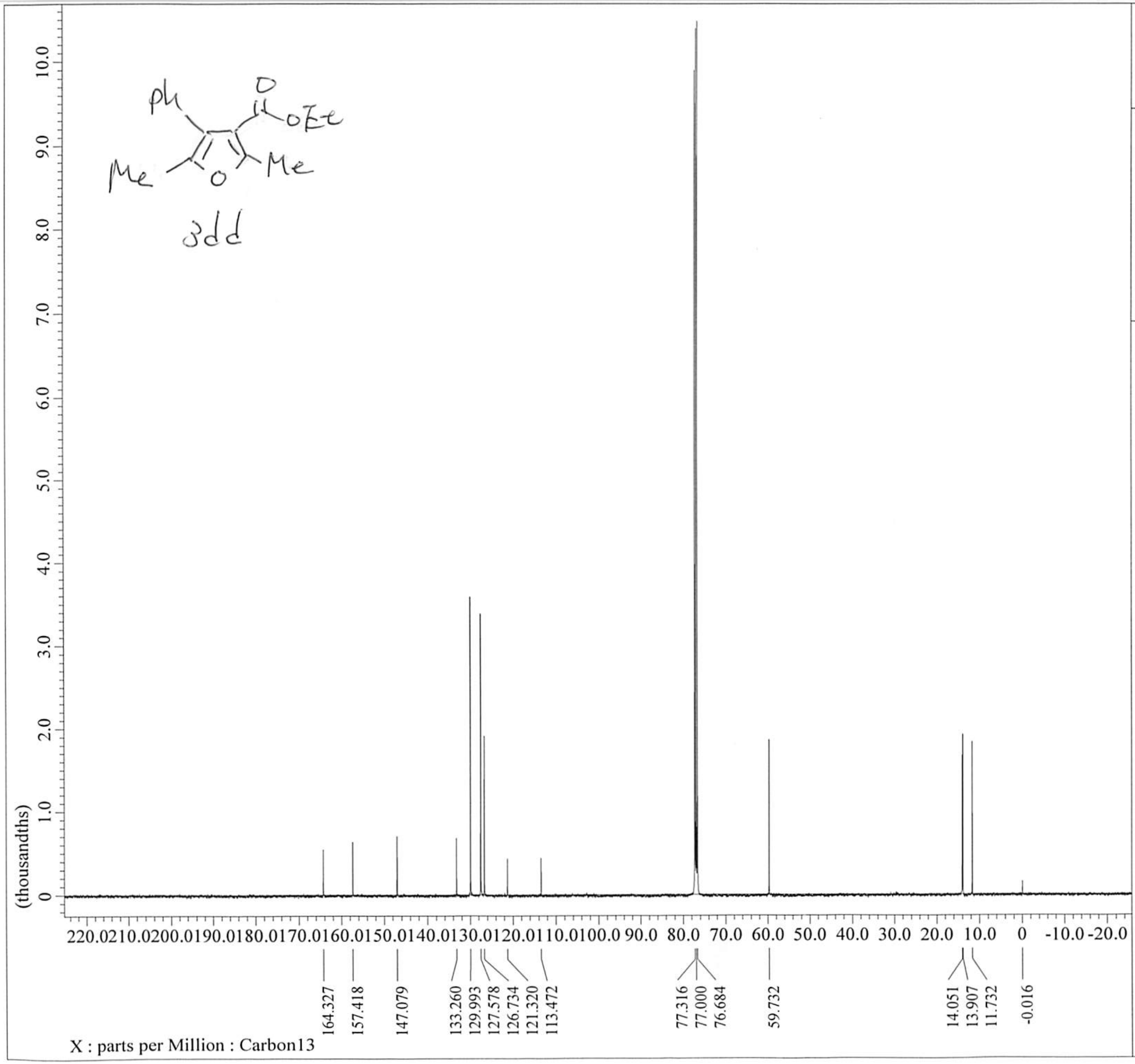


```
---- PROCESSING PARAMETERS ----
sexp( 2.0[Hz], 0.0[s] )
trapezoid( 0[%], 0[%], 80[%], 100[%] )
zerofill( 1 )
fft( 1, TRUE, TRUE )
machinephase
ppm

以下に由来:: chiaki_7_1_carbon-1-1.jdf
```

```
Filename                    = chiaki_7_1_carbon-
Author                      = delta
Experiment                  = carbon.jxp
Sample_Id                   = chiaki_7_1
Solvent                     = CHLOROFORM-D
Creation_Time               = 12-FEB-2020 21:02:
Revision_Time               = 13-FEB-2020 15:08:
Current_Time                = 13-FEB-2020 15:09:

Comment                     = HRD173 cd2cl2 p30
Data_Format                 = 1D COMPLEX
Dim_Size                    = 26214
Dim_Title                   = Carbon13
Dim_Units                   = [ppm]
Dimensions                  = X
Spectrometer                = JNM-ECZ400S/L1

Field_Strength              = 9.389766[T] (400[M
X_Acq_Duration              = 1.03809024[s]
X_Domain                    = 13C
X_Freq                      = 100.52530333[MHz]
X_Offset                    = 100[ppm]
X_Points                    = 32768
X_Prescans                  = 4
X_Resolution                = 0.96330739[Hz]
X_Sweep                     = 31.56565657[kHz]
X_Sweep_Clipped             = 25.25252525[kHz]
Irr_Domain                  = Proton
Irr_Freq                    = 399.78219838[MHz]
Irr_Offset                  = 5[ppm]
Blanking                    = 5[us]
Clipped                     = FALSE
Decimation_Reg              = r: 198( 197),g: 39
Scans                       = 8192
Total_Scans                 = 8192

Relaxation_Delay            = 2[s]
Recvr_Gain                  = 50
Temp_Get                    = 20.8[dC]
X_90_Width                  = 9.7[us]
X_Acq_Time                  = 1.03809024[s]
X_Angle                     = 30[deg]
X_Atn                       = 8.2[dB]
X_Pulse                     = 3.23333333[us]
Irr_Atn_Dec                 = 28.124[dB]
Irr_Atn_Dec_Calc            = 28.124[dB]
Irr_Atn_Dec_Default_Calc    = 28.124[dB]
Irr_Atn_Noe                 = 28.124[dB]
Irr_Dec_Bandwidth_Hz        = 4.7826087[kHz]
Irr_Dec_Bandwidth_Ppm       = 11.96303566[ppm]
```

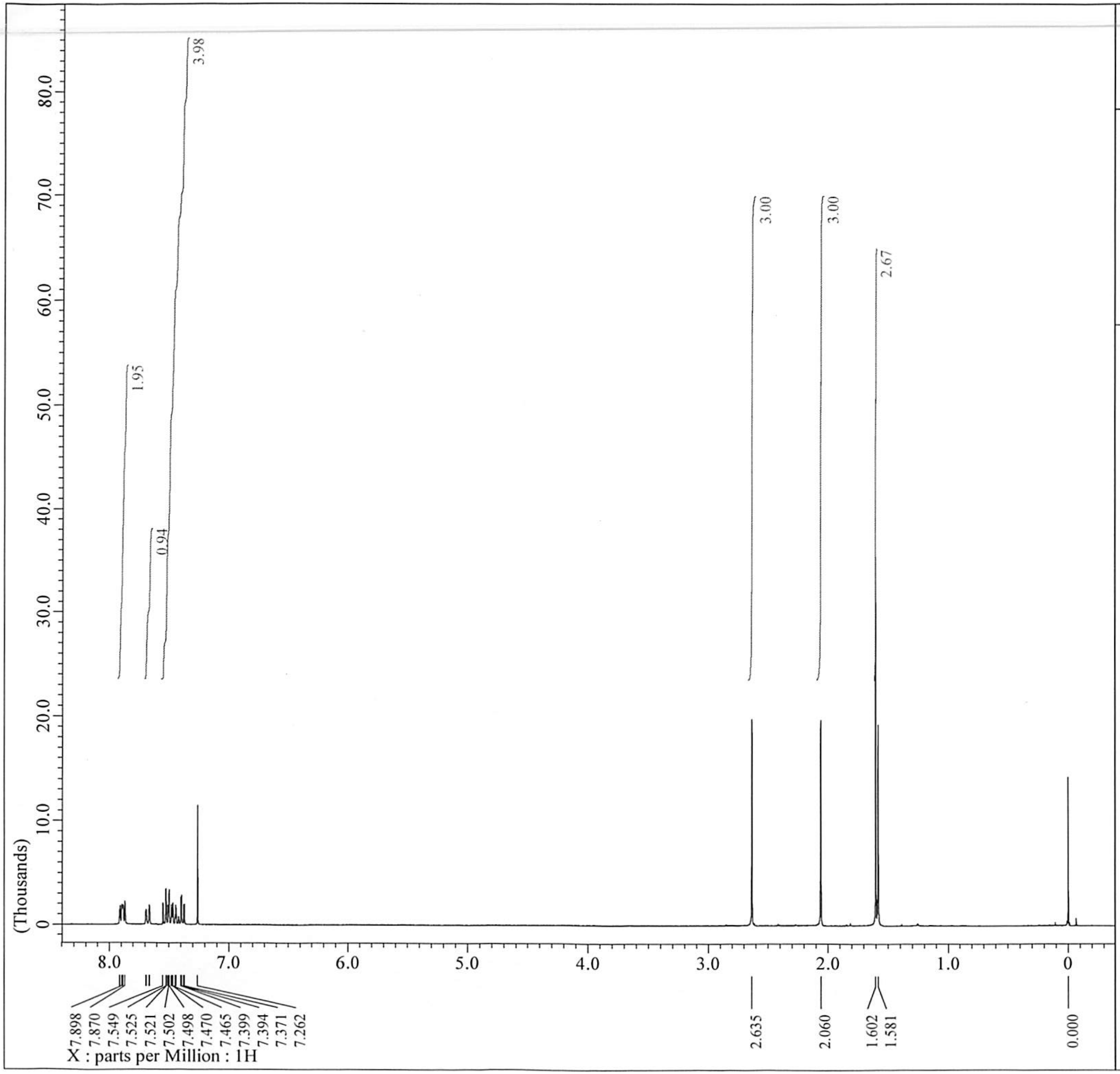

JEOL
Solutions for Innovation

```
---- PROCESSING PARAMETERS ----
dc_balance( 0, FALSE )
sexp( 0.2[Hz], 0.0[s] )
trapezoid( 0[%], 0[%], 80[%], 100[%] )
zerofill( 1 )
fft( 1, TRUE, TRUE )
machinephase
ppm

以下に由来:: Morita_2830-1.jdf
```

```
Filename         = Morita_2830-7.jdf
Author           = Administrator
Experiment       = zg30
Sample_Id        = kT24-Fr10
Solvent          = CHLOROFORM-D
Creation_Time    = 26-AUG-2022 17:25:28
Revision_Time    = 14-NOV-2023 18:48:49
Current_Time     = 14-NOV-2023 18:49:00

Comment          = kT24-Fr10
Data_Format      = 1D COMPLEX
Dim_Size         = 32768
Dim_Title        = 1H
Dim_Units        = [ppm]
Dimensions       = X
Spectrometer     = BRUKER_DMX_NMR

Field_Strength = 7.0492145[T] (300[MHz])
X_Domain         = 1H
X_Freq           = 300.13185343[MHz]
X_Freq_Flip      = TRUE
X_Offset         = 1.85342561[kHz]
X_Points         = 32768
X_Prescans       = 2
X_Sweep          = 6.18811881[kHz]
Scans            = 16

Temp_Get         = 294.76[K]
Filter_Factor    = 3232
```

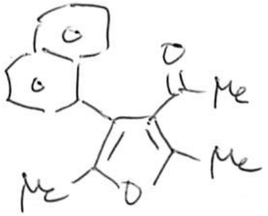

3ea

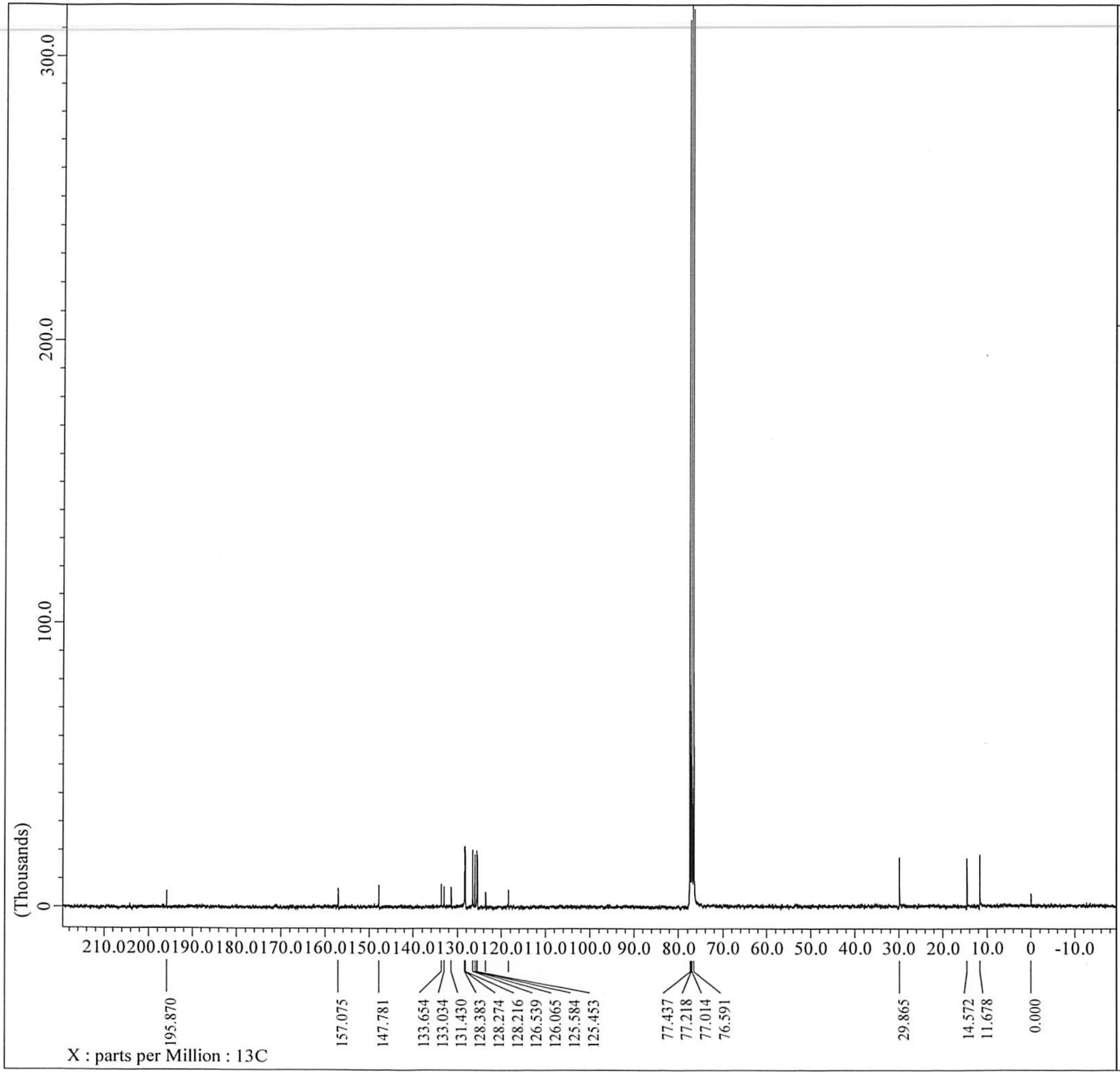

JEOL
Solutions for Innovation

```
---- PROCESSING PARAMETERS ----
dc_balance( 0, FALSE )
sexp( 2.0[Hz], 0.0[s] )
trapezoid( 0[%], 0[%], 80[%], 100[%] )
zerofill( 1 )
fft( 1, TRUE, TRUE )
machinephase
ppm
phase( -5, -15, 50[%] )

以下に由来:: Morita_2850-1.jdf
```

```
Filename        = Morita_2850-4.jdf
Author          = Administrator
Experiment      = zgpg30
Sample_Id       = KY24_Fr10_13CNMR
Solvent         = CHLOROFORM-D
Creation_Time   = 26-AUG-2022 17:27:53
Revision_Time   = 14-NOV-2023 18:53:40
Current_Time    = 14-NOV-2023 18:53:56

Comment         = KY24_Fr10_13CNMR
Data_Format     = 1D COMPLEX
Dim_Size        = 32768
Dim_Title       = 13C
Dim_Units       = [ppm]
Dimensions      = X
Spectrometer    = BRUKER_DMX_NMR

Field_Strength  = 7.0492145[T] (300[MHz])
X_Domain        = 13C
X_Freq          = 75.4752953[MHz]
X_Freq_Flip     = TRUE
X_Offset        = 7.54630085[kHz]
X_Points        = 32768
X_Prescans      = 4
X_Sweep         = 18.02884615[kHz]
Scans           = 10000

Temp_Get        = 296.86[K]
Filter_Factor   = 1109
```

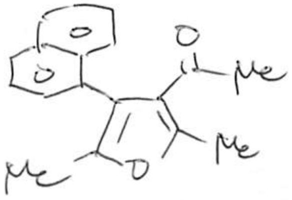

3ea

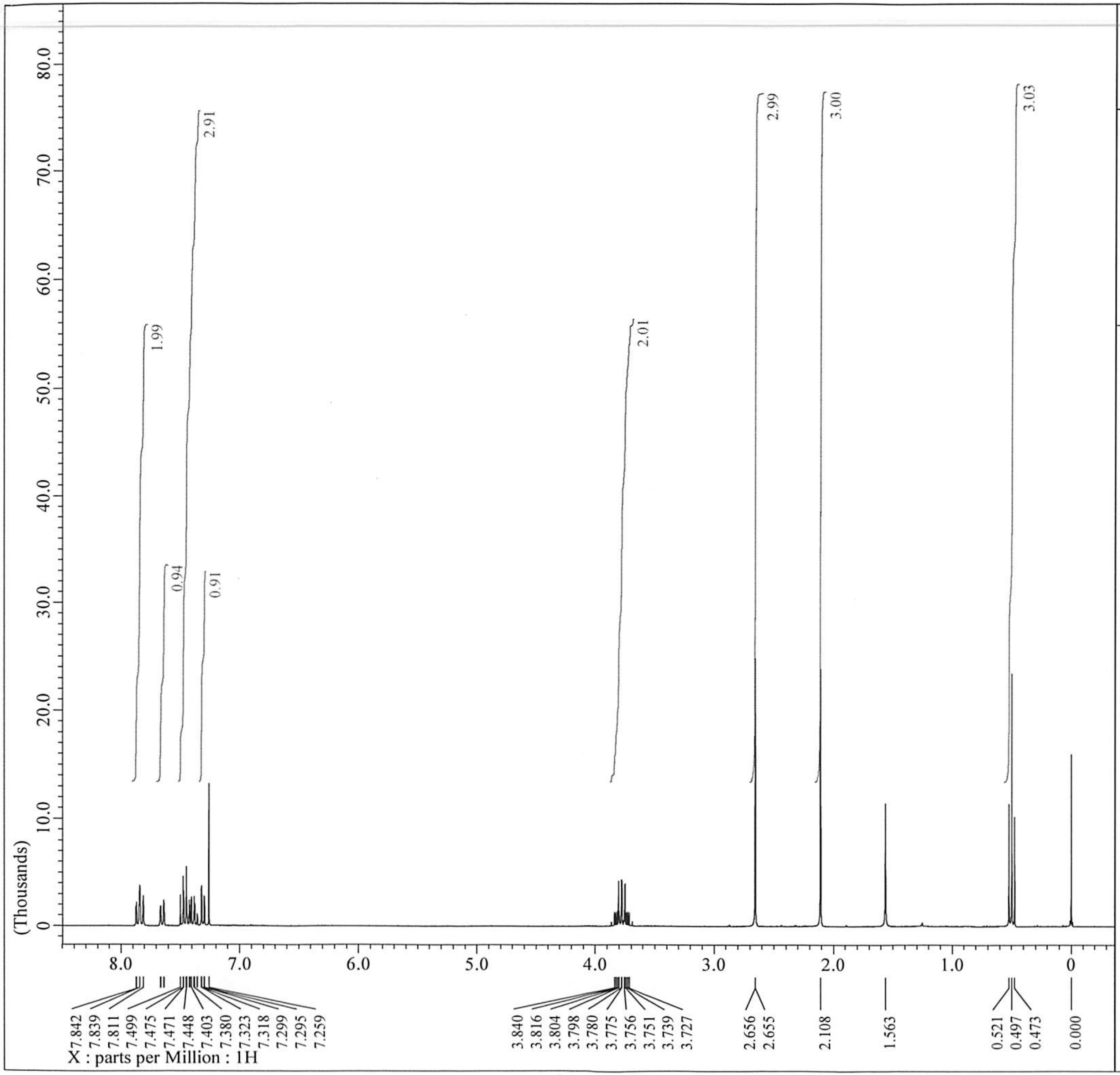

JEOL
Solutions for Innovation

```
---- PROCESSING PARAMETERS ----
dc_balance( 0, FALSE )
sexp( 0.2[Hz], 0.0[s] )
trapezoid( 0[%], 0[%], 80[%], 100[%] )
zerofill( 1 )
fft( 1, TRUE, TRUE )
machinephase
ppm
phase( 0, 15, 50[%] )

以下に由来:: Morita_2800-6.jdf
```

```
Filename        = Morita_2800-10.jdf
Author          = Administrator
Experiment      = zg30
Sample_Id       = KT22-Fr8
Solvent         = CHLOROFORM-D
Creation_Time   = 26-AUG-2022 17:17:10
Revision_Time   = 14-NOV-2023 18:31:26
Current_Time    = 14-NOV-2023 18:31:47

Comment         = KT22-Fr8
Data_Format     = 1D COMPLEX
Dim_Size        = 32768
Dim_Title       = 1H
Dim_Units       = [ppm]
Dimensions      = X
Spectrometer    = BRUKER_DMX_NMR

Field_Strength  = 7.0492145[T] (300[MHz])
X_Domain        = 1H
X_Freq          = 300.13185343[MHz]
X_Freq_Flip     = TRUE
X_Offset        = 1.85342561[kHz]
X_Points        = 32768
X_Prescans      = 2
X_Sweep         = 6.18811881[kHz]
Scans           = 16

Temp_Get        = 294.86[K]
Filter_Factor   = 3232
```

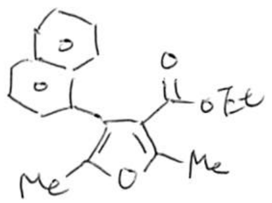

3ec

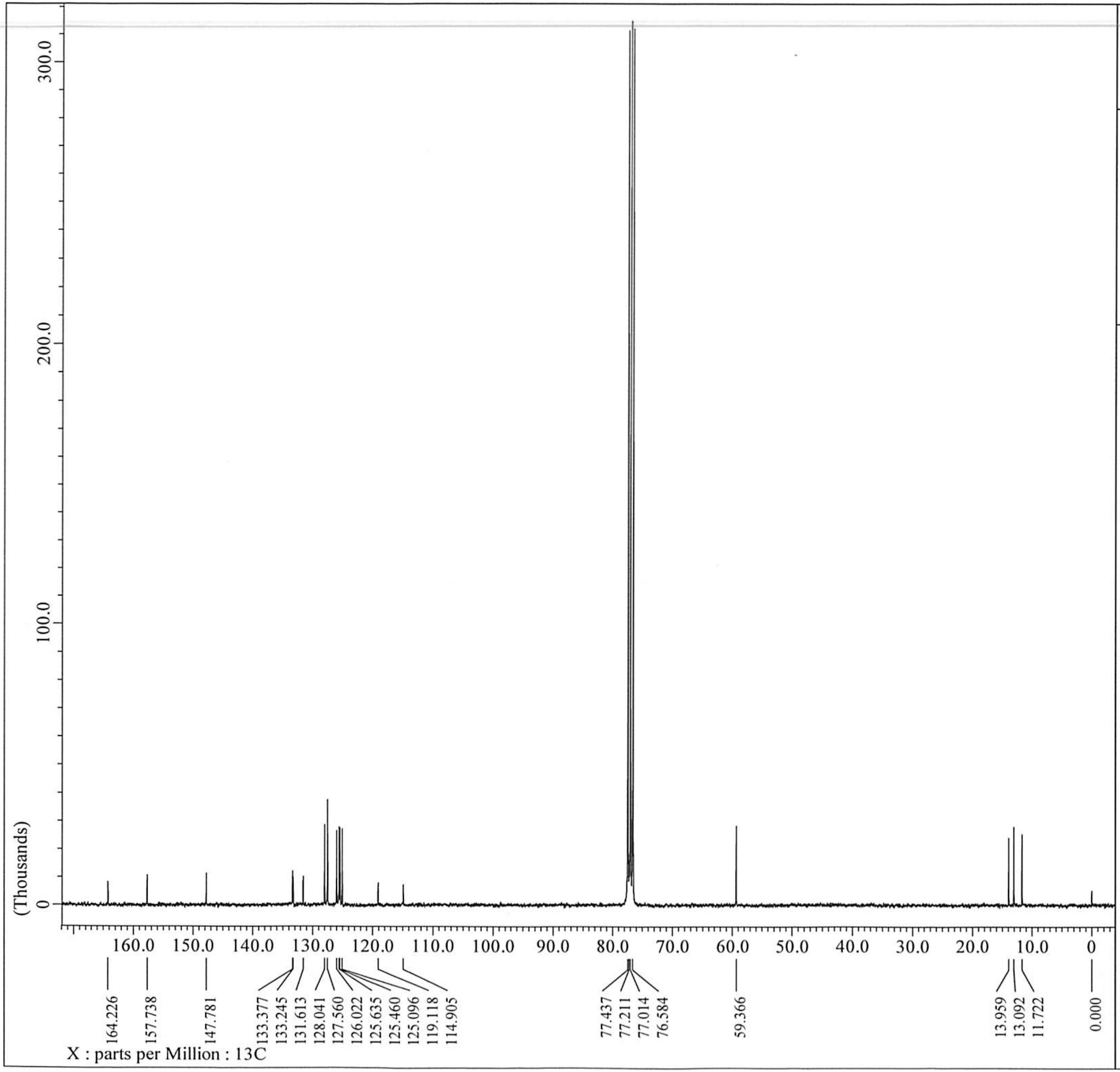

JEOL
Solutions for Innovation

---- PROCESSING PARAMETERS ----
dc_balance(0, FALSE)
sexp(2.0[Hz], 0.0[s])
trapezoid(0[%], 0[%], 80[%], 100[%])
zerofill(1)
fft(1, TRUE, TRUE)
machinephase
ppm

以下に由来:: Morita_2820-4.jdf

Filename	=	Morita_2820-6.jdf
Author	=	Administrator
Experiment	=	zgpg30
Sample_Id	=	KT22_Fr8_13C
Solvent	=	CHLOROFORM-D
Creation_Time	=	26-AUG-2022 17:23:40
Revision_Time	=	14-NOV-2023 18:57:42
Current_Time	=	14-NOV-2023 18:57:59
Comment	=	KT22_Fr8_13C
Data_Format	=	1D COMPLEX
Dim_Size	=	32768
Dim_Title	=	13C
Dim_Units	=	[ppm]
Dimensions	=	X
Spectrometer	=	BRUKER_DMX_NMR
Field_Strength	=	7.0492145[T] (300[MHz])
X_Domain	=	13C
X_Freq	=	75.4752953[MHz]
X_Freq_Flip	=	TRUE
X_Offset	=	7.54630085[kHz]
X_Points	=	32768
X_Prescans	=	4
X_Sweep	=	18.02884615[kHz]
Scans	=	10000
Temp_Get	=	296.86[K]
Filter_Factor	=	1109

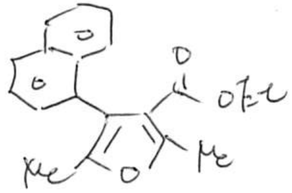

3ec

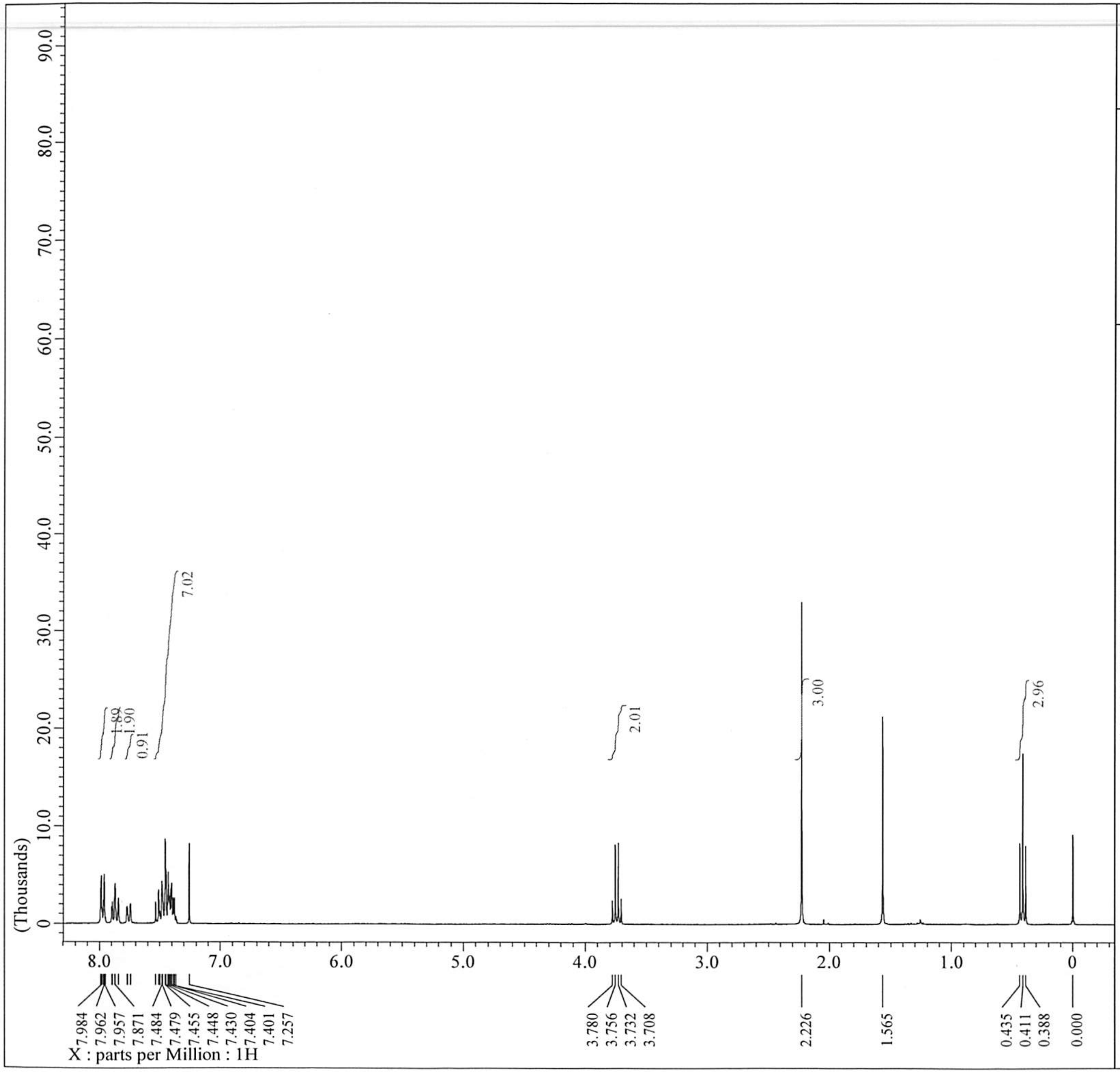

JEOL
Solutions for Innovation

---- PROCESSING PARAMETERS ----
dc_balance(0, FALSE)
sexp(0.2[Hz], 0.0[s])
trapezoid(0[%], 0[%], 80[%], 100[%])
zerofill(1)
fft(1, TRUE, TRUE)
machinephase
ppm

以下に由来:: Morita_2870-1.jdf

Filename	=	Morita_2870-5.jdf
Author	=	Administrator
Experiment	=	zg30
Sample_Id	=	Kt25-Fr7
Solvent	=	CHLOROFORM-D
Creation_Time	=	26-AUG-2022 17:35:47
Revision_Time	=	14-NOV-2023 18:41:09
Current_Time	=	14-NOV-2023 18:41:38
Comment	=	Kt25-Fr7
Data_Format	=	1D COMPLEX
Dim_Size	=	32768
Dim_Title	=	1H
Dim_Units	=	[ppm]
Dimensions	=	X
Spectrometer	=	BRUKER_DMX_NMR
Field_Strength	=	7.0492145[T] (300[MHz])
X_Domain	=	1H
X_Freq	=	300.13185343[MHz]
X_Freq_Flip	=	TRUE
X_Offset	=	1.85342561[kHz]
X_Points	=	32768
X_Prescans	=	2
X_Sweep	=	6.18811881[kHz]
Scans	=	16
Temp_Get	=	294.86[K]
Filter_Factor	=	3232

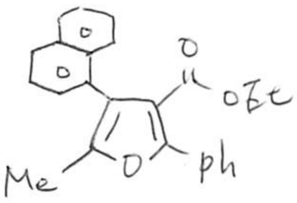

3ed

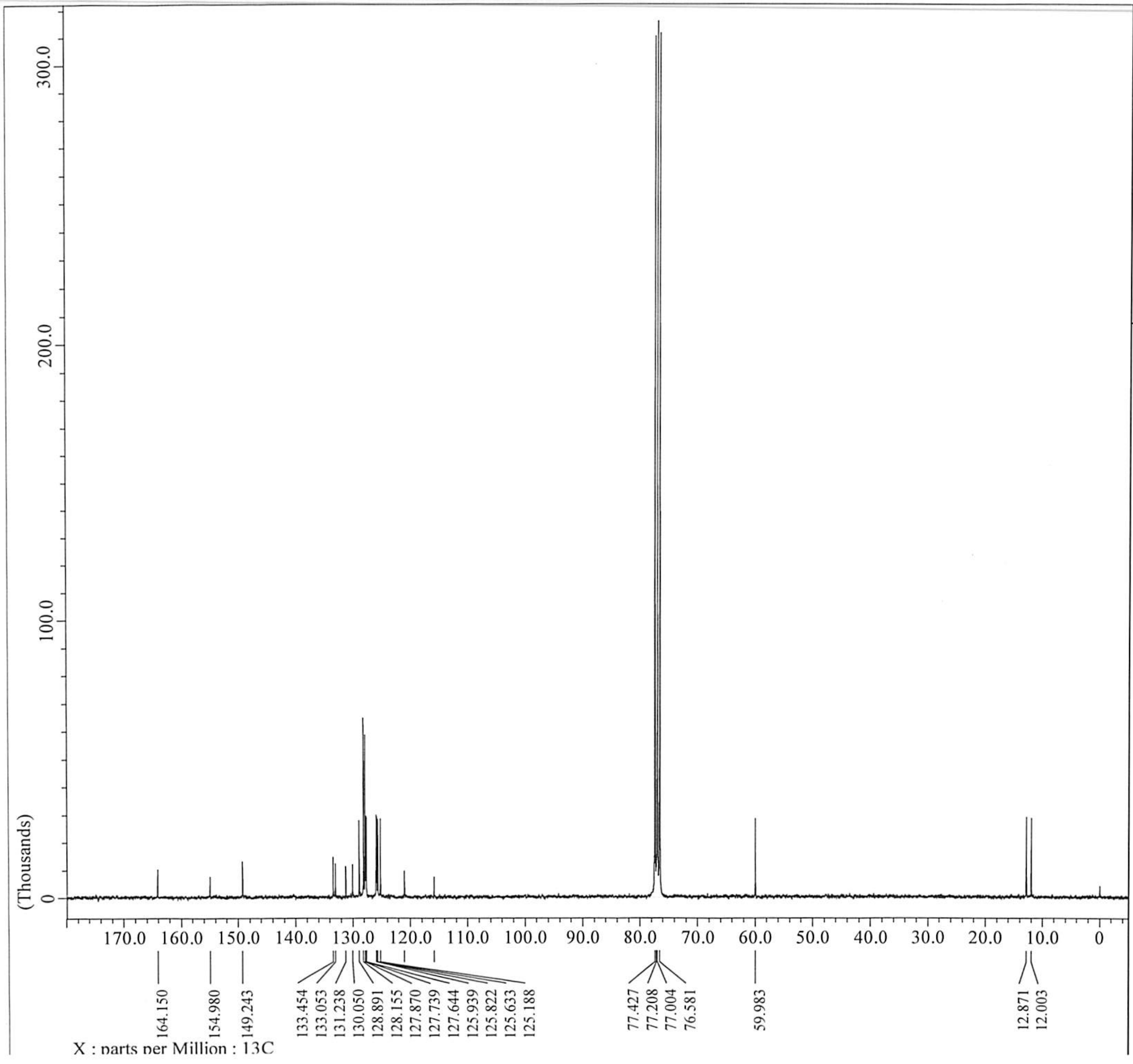

JEOL
Solutions for Innovation

```
---- PROCESSING PARAMETERS ----
dc_balance( 0, FALSE )
sexp( 2.0[Hz], 0.0[s] )
trapezoid( 0[%], 0[%], 80[%], 100[%] )
zerofill( 1 )
fft( 1, TRUE, TRUE )
machinephase
ppm

以下に由来:: Morita_2880-1.jdf
```

```
Filename        = Morita_2880-2.jdf
Author          = Administrator
Experiment      = zgpg30
Sample_Id       = KT25_fr7-13CNMR
Solvent         = CHLOROFORM-D
Creation_Time   = 29-AUG-2022 11:55:29
Revision_Time   = 29-AUG-2022 11:55:37
Current_Time    = 29-AUG-2022 11:56:05

Comment         = KT25_fr7-13CNMR
Data_Format     = 1D COMPLEX
Dim_Size        = 32768
Dim_Title       = 13C
Dim_Units       = [ppm]
Dimensions      = X
Spectrometer    = BRUKER_DMX_NMR

Field_Strength  = 7.0492145[T] (300[MHz])
X_Domain        = 13C
X_Freq          = 75.4752953[MHz]
X_Freq_Flip     = TRUE
X_Offset        = 7.54630085[kHz]
X_Points        = 32768
X_Prescans      = 4
X_Sweep         = 18.02884615[kHz]
Scans           = 10000

Temp_Get        = 296.96[K]
Filter_Factor   = 1109
```

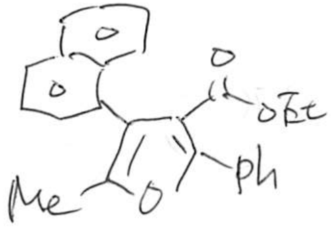

3ed

161220NM525

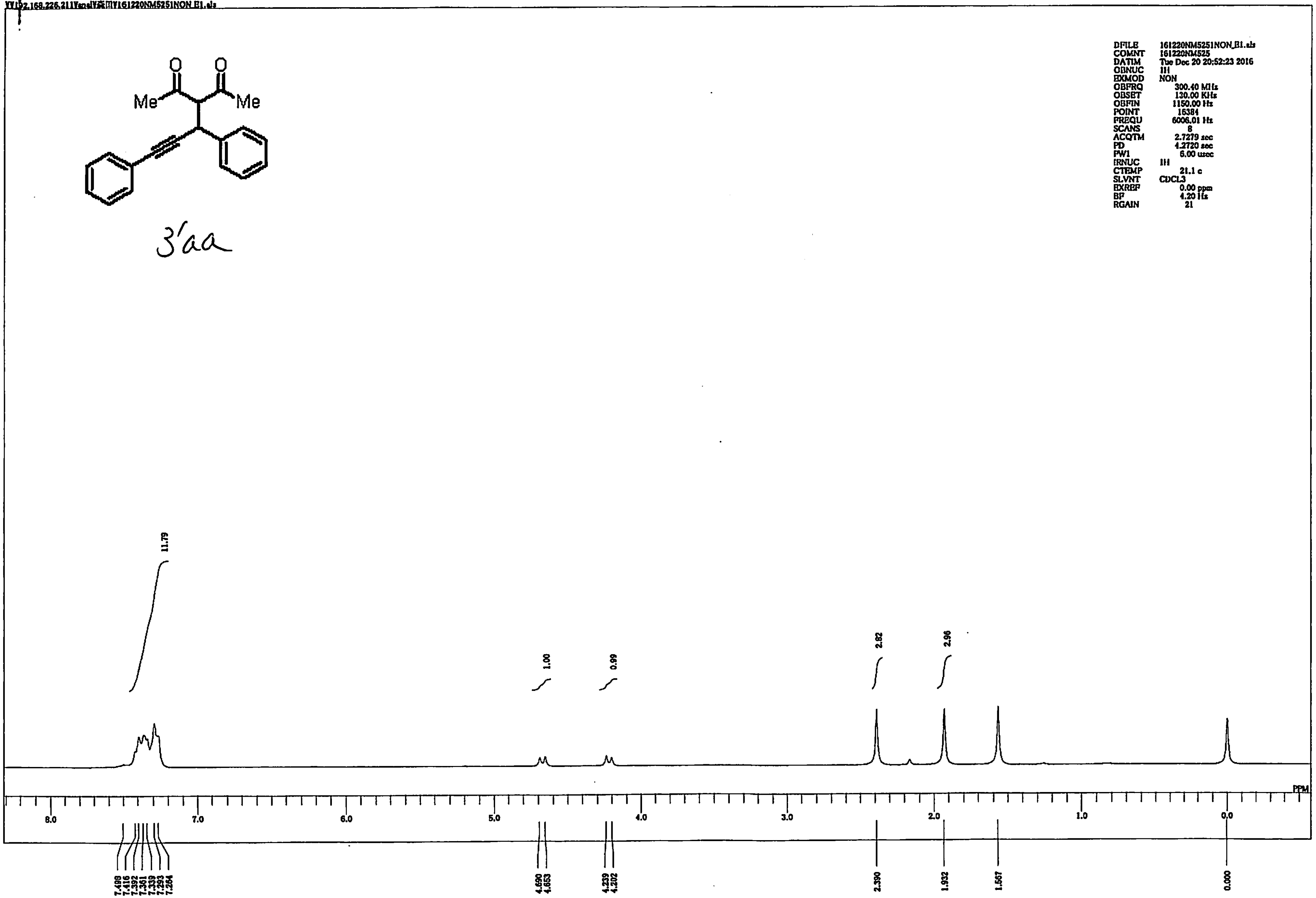

161221NM525

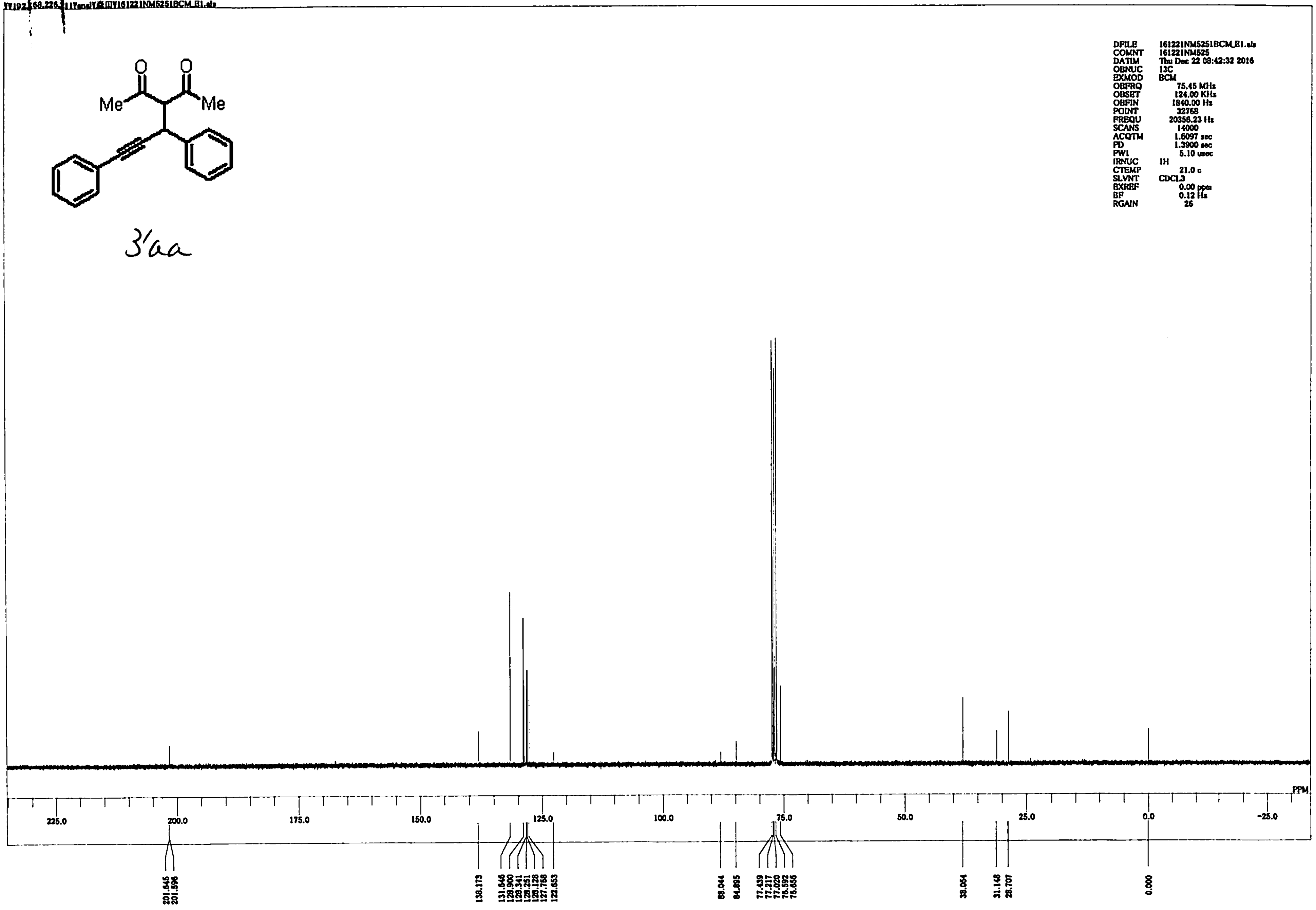

Supplement: Supplementary file 1 [file molecules-29-00378-s001.zip › molecules-2774763-supplementary.pdf]
